# Design, synthesis, and molecular docking studies of novel pomalidomide-based PROTACs as potential anti-cancer agents targeting EGFR^WT^ and EGFR^T790M^

**DOI:** 10.1080/14756366.2022.2062338

**Published:** 2022-04-26

**Authors:** Moustafa O. Aboelez, Amany Belal, Guangya Xiang, Xiang Ma

**Affiliations:** a School of Pharmacy, Tongji Medical College, Huazhong University of Science and Technology, Wuhan, China; b Department of Pharmaceutical Chemistry, Faculty of Pharmacy, Sohag University, Sohag, Egypt; c Department of Pharmaceutical Chemistry, College of Pharmacy, Taif University, Taif, Saudi Arabia

**Keywords:** EGFR, PROTACs, pomalidomide, apoptosis induction, anticancer agents

## Abstract

A new class of EGFR PROTACs based on pomalidomide was developed, synthesised, and tested for their cytotoxic activity against a panel of human cancer cells. Compounds **15**–**21** were showed to be more effective against the four tested cell lines than erlotinib. In particular, compound **16** was found to be the most potent counterpart as it was 5.55, 4.34, 5.04, and 7.18 times more active than erlotinib against MCF-7, HepG-2, HCT-116, and A549 cells, respectively. Compound **15** was revealed to be more active than doxorubicin against the four tested cell lines. Furthermore, the most potent cytotoxic compounds were studied further for their kinase inhibitory effects against EGFR^WT^ and EGFR^T790M^ using HTRF test. Compound **16** showed to be the most effective against both kinds of EGFR, with IC_50_ values of 0.10 and 4.02 µM, respectively. Compound **16** could effectively degrade EGFR protein through ubiquitination (D_max_ = 96%) at 72 h in the tested cells.

## Graphical Abstract

**Figure:**
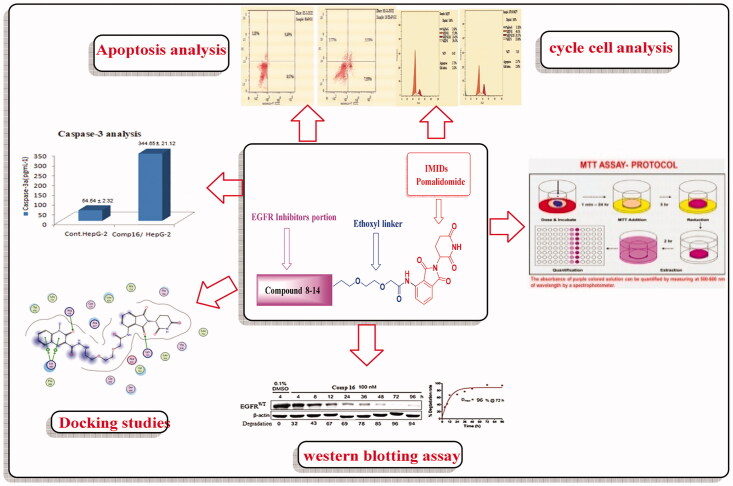

## 1. Introduction

The epidermal growth factor receptor (EGFR) is a transmembrane tyrosine kinase protein that regulates cell proliferation, invasion, metastasis, and apoptosis in human epithelial cells by acting as a receptor for members of the EGF family.^1–3^ EGFR gene amplification has been associated to several human malignancies, including oesophageal cancer, glioblastoma, anal cancers, malignancies of the epithelium of the head and neck, breast cancers, and lung cancers, particularly non-small-cell lung cancers (NSCLCs).^3–6^

Mutations in the EGFR kinase adenosine triphosphate (ATP)-binding domain is considered as the oncogenic driver in NSCLC, such as in-frame deletions of exon 19 and the L858R mutation.^1^ Lung cancer is the most common cancer related to death in the world, and NSCLCs are one of the most common types of lung cancer.^7^ The biomedical community has actively examined EGFR as a therapeutic target for NSCLC. Studies on inhibiting the activity of mutant EGFR ATP-binding domains resulted in the creation of a variety of FDA-approved EGFR tyrosine kinase inhibitors (TKIs). In NSCLC patients, the first-generation of TKIs, gefitinib^8^ and erlotinib,^9^ exhibited significant responses and prolonged survival rates. The secondary "gatekeeper" T790M mutation, on the other hand, enhanced ATP-binding affinity and induced recurrence in the majority of NSCLC patients after 9–14 months of treatment.^10–14^

To prevent resistance, second-generation of EGFR inhibitors have been developed, including afatinib and dacomitinib, which target EGFR with the T790M activating mutation. Following that, the third-generation of EGFR covalent inhibitors was created, with increased selectivity to (WT) EGFR^13^. However, acquired resistance to irreversible EGFR-TKIs has been linked to the C797S point mutation and/or other mechanisms, making NSCLC resistant to these inhibitors.^15–19^

The fourth-generation EGFR-TKIs, such as EAI045^20^ and other noncovalent inhibitors targeting allosteric binding site (s), appears to be a substantial break through against these tertiary mutations.^21–23^ Despite this progress, there is still a medical need for new small-molecule inhibitors or therapeutic methods to overcome multipoint EGFR mutations.^24^

Proteolysis targeting chimaeras (PROTACs) can target a specific protein for degradation as a potential therapeutic method.^25–30^ PROTAC-induced proximity causes preferential polyubiquitination of the target protein, which leads to proteasome destruction. Unlike typical enzyme inhibitors, which limit the target enzyme’s catalytic activity, PROTACs cause the target protein to degrade. As a result, the new bifunctional small-molecule-mediated protein degradation paradigm has the potential to overcome the disadvantages of traditional occupancy-driven inhibitors. This method has been successfully used for the degradation of a variety of proteins in recent years^31^.

**Figure 1.**
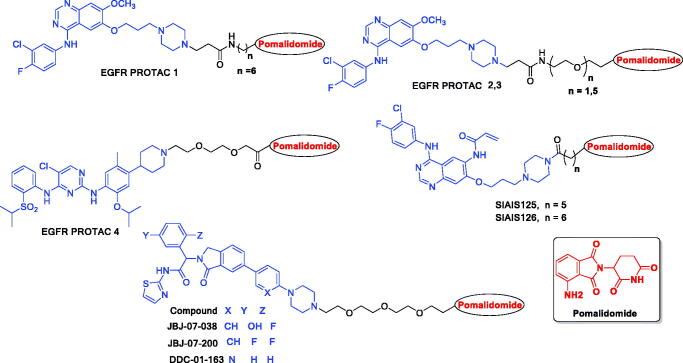
Chemical structures of previously published EGFR targeting PROTACs based on pomalidomide.

Meng *et al*. developed a set of putative EGFR degraders (**EGFR PROTAC 1–3**) in order to investigate a potential novel therapeutic strategy for NSCLC and overcome drug resistance (Figure 1). As shown in (Figure 1), two new CRBN-based EGFR targeting PROTACs (**SIAIS125** and **SIAIS126**) were reported to induce degradation for both EGFR^Ex19del^ and EGFR*^L858R/T790M^* resistant proteins. It is noticeable from their chemical structures that they are based on pomalidomide. Additionally there are EGFR targeting small molecule PROTACs based also on pomalidomide (Figure 1) that have showed selective and potent antitumor activities in EGFR-TKI resistant lung cancer cells and can stimulate necrosis and stop cell cycle in H1975 cells.^31^ All these facts encouraged us to design a new pomalidomide based EGFR targeting PROTACs to get new hopeful candidates that can affect both types of EGFR either wild or mutant.

**Figure 2.**
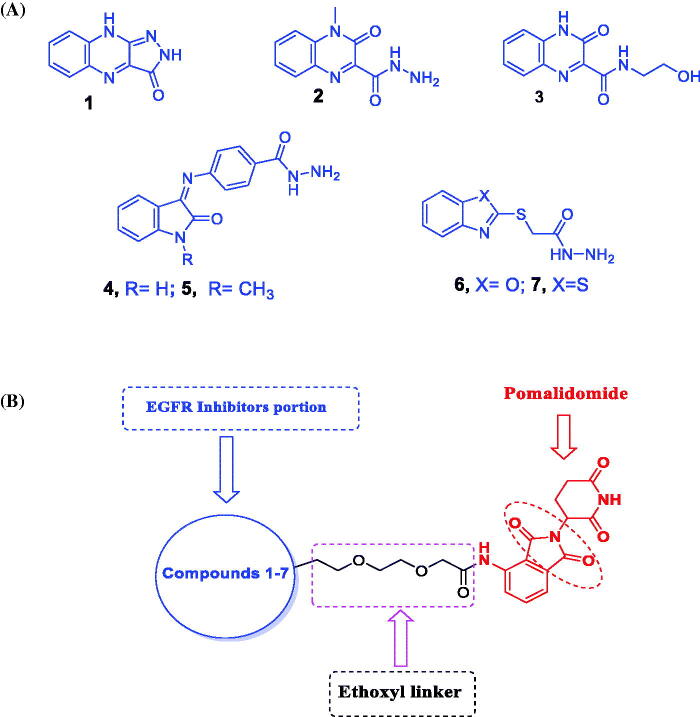
(A) Chemical structures of some reported EGFR-TK inhibitors. (B) Design of EGFR-targeting PROTACs in this work.

In a previous study, a variety of EGFR-TKI inhibitors were developed,^32–36^ which are fused heterocycles with a quinoxaline moiety **1–3** (Figure 2(A)). Those inhibitors have good anti-proliferative effects against breast cancer (MCF-7), hepatocellular carcinoma (HepG-2), colorectal carcinoma (HCT-116), and non-small cell lung cancer cells (A549) and exhibited EGFR inhibitory action*^32^*. Additionally, when compared to erlotinib, compounds **4–7** (Figure 2(A)), demonstrated substantial EGFR inhibitory action, and these findings are consistent with our docking studies.

These findings imply that these compounds with EGFR inhibitory action could be useful in anticancer treatments. We used compounds **1–7** as ligands of EGFR to try to find a new EGFR degrader. The design, synthesis, and bioactivity evaluation of dioxopiperidinyl moiety derivatives **15–21** as new EGFR degraders were described in this study. The CRBN ligand pomalidomide and the VHL ligand were chosen as E3 ligase-recruiting components in our EGFR-targeting PROTACs (Figure 2(B)).

## 2. Results and discussion

### 2.1. Design and synthesis of EGFR-targeting PROTACs

**Scheme 1.**
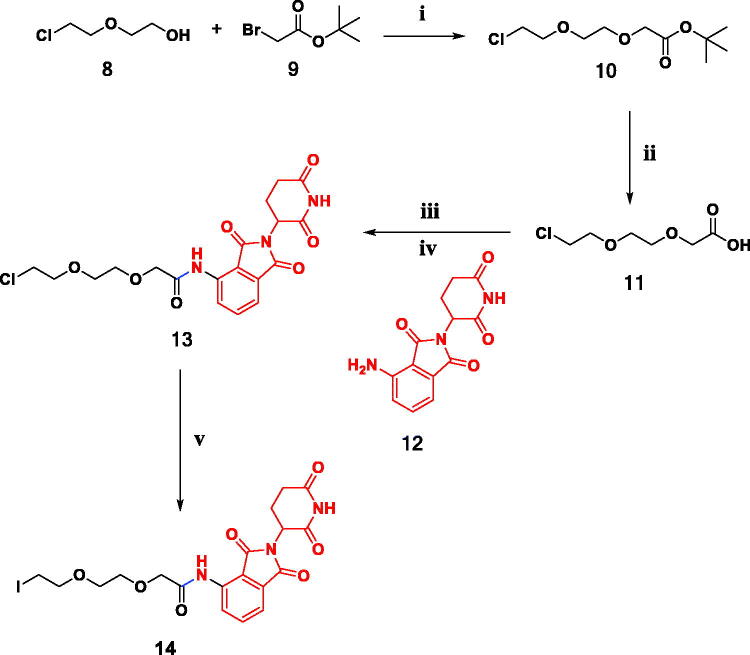
Synthesis of key intermediate **14**. Reagents and conditions: (i) t-BuOK, THF, stirring, overnight, r.t.; (ii) 20% THF\DCM, r.t., 1 hr; (iii) SOCl_2_, DCM, stirring, r.t., 2 hr; (iv) THF, DIPEA, reflux, 8 h; (v) NaI, Acetone, reflux, overnight.

**Scheme 2.**
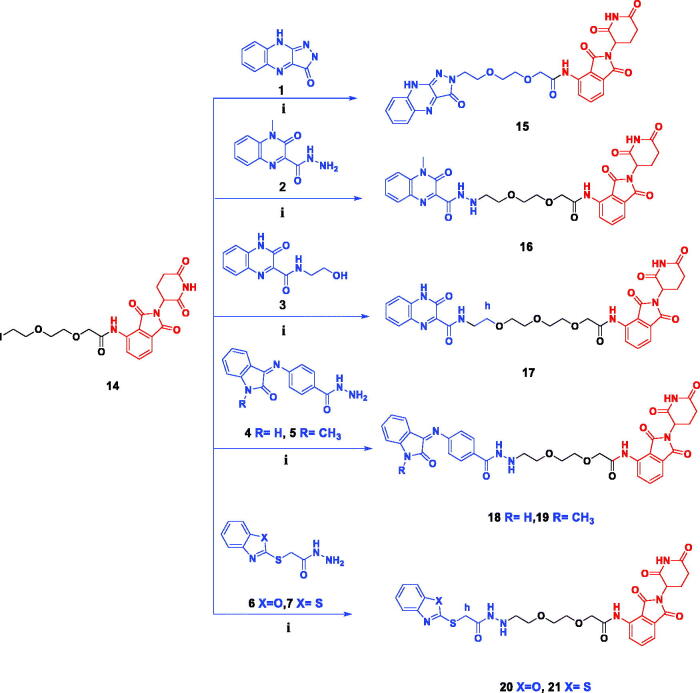
Synthesis of target compounds **15–21**. Reagents and conditions: (i) DIPEA, DMF, reflux, overnight.

Pomalidomide (4-amino-2-(2,6-dioxopiperodin-3-yl)-isoindoles-1,3-dione) was used as an E3 ubiquitin ligase ligand,^28^ the second generation of immuno-modulatory drugs (IMIDs), and has a higher cellular stability than other IMIDs.^29^^,^^37^ The synthetic methods used to prepare the designed compounds **15–21** were depicted in Schemes 1 and 2. We aimed to build up *N*-(2-(2,6-dioxopiperidin-3-yl)-1,3-dioxoisoindolin-4-yl)-2-(2-iodoethoxy)ethoxy)acetamide **14** by "expanding" the linker attached to the pomalidomide end **12,**^38^ hydrolysis of *tert* butyl 2-(2-(2-chloroethoxy)ethoxy)acetate **10** with trifluroacetic acid (TFA) in dichloromethane (DCM) yielded 2-(2-(2-chloroethoxy)ethoxy)acetic acid **11.**^38^ Then, compound **11** was activated to acyl chloride *via* its reaction with thionyl chloride in tetrahydrofuran (THF) and then subjected to react with pomalidomide (**12)** in presence of diisopropylethylamine (DIPEA) as a base to afford 2-(2-(2-chloroethoxy)ethoxy)-*N*-(2-(2,6-dioxopiperidin-3-yl)-1,3-dioxoisoindolin-4-yl)acetamide **13**^38^ (Scheme 1).

Compound **13** was converted to *N*-(2-(2,6-dioxopiperidin-3-yl)-1,3-dioxoisoindolin-4-yl)-2-(2-iodoethoxy)ethoxy) acetamide (**14)** *via* a Finkelstein reaction^38^ (Scheme 1). Compound **14** was used as a key material to create target compounds **15**–**21** in acceptable yields *via* nucleophilic substitution reaction with quinoxalines **1–3**, isatin hydrazides **4,5**, benzoxazole hydrazide **6** and benzothiazole hydrazide **7** respectively^32–36^ (Scheme 2).

Target compounds **15, 16** and **17** were obtained (56–60% yields, purification by flash chromatography) through the reaction of compound **14** with quinoxalines **1–3** by heating under reflux at 80 °C overnight, using methyl-2-pyrrolidone (NMP) as solvent and DIPEA as a base.

Synthesised compounds **18** and **19** (52**–**56% yield, purification by flash chromatography), were obtained by the same previous method *via* the treatment of intermediate key **14** with isatin hydrazides **4** and/or **5,** respectively.

Furthermore, the reaction of compound **14** with benzoxazole hydrazide **6** and benzothiazole hydrazide **7**, resulted in the formation of target compounds **20** and **21**, respectively (50**–**52% yield, purification by flash chromatography) by the same previous method (Scheme 2). Proposed structures for EGFR PROTACs degraders **15–21** were in agreement with their various spectroscopic and analytical data (Supplementary data files).

### 2.2. Biological evaluations

#### 2.2.1. In vitro *cytotoxic activities*

The anti-proliferative activity *in vitro* of target compounds **15–21** against a panel of four cell lines MCF-7, HepG-2, HCT-116 and A549 were evaluated using MTT assay.^39^^,^^40^ Erlotinib and doxorubicin were applied in the experiments as references. In MCF-7, HepG-2 and A549 cell lines, EGFR^WT^ is overexpressed.^41–43^ The results were illustrated in (Table 1) as IC_50_ (µM). Compounds **15–21** showed to be more active against the four tested cell lines than erlotinib. In particular, compound **16** that showed to be 5.55, 4.34, 5.04 and 7.18 folds more active than erlotinib in MCF-7, HepG-2, HCT-116 and A549 cells, respectively. Compound **16** was also more effective than doxorubicin against MCF-7, HepG-2 and HCT-116 cells, although compound **15** was more effective against MCF-7 and HepG-2 cells.

**Table 1.** *In vitro* anti-proliferative activities of target compounds **15–21** against MCF-7, HepG-2, HCT-116 and A549 cells line.

Compounds	IC_50_ (µM)^a^			
	MCF-7	HepG-2	HCT-116	A549
**15**	7.87 ± 0.31	3.89 ± 0.05	6.19 ± 0.27	3.09 ± 0.15
**16**	3.92 ± 0.19	3.02 ± 0.12	3.32 ± 0.15	2.69 ± 0.09
**17**	7.96 ± 0.35	4.09 ± 0.11	6.29 ± 0.35	3.19 ± 0.19
**18**	8.26 ± 0.25	6.26 ± 0.56	6.79 ± 0.27	4.67 ± 0.13
**19**	16.26 ± 0.71	8.26 ± 0.36	9.12 ± 0.38	6.54 ± 0.32
**20**	14.26 ± 0.69	9.26 ± 0.39	12.26 ± 0.71	8.27 ± 0.25
**21**	16.26 ± 0.71	9.86 ± 0.37	12.96 ± 0.52	7.27 ± 0.16
**Erlotinib**	21.76 ± 1.85	13.11 ± 1.28	16.76 ± 1.65	19.33 ± 1.85
**Doxorubicin**	7.89 ± 0.55	6.22 ± 0.45	5.52 ± 0.25	NT

^a^IC_50_ values are the mean ± S.D of three experiments.

NT: Compounds not investigated.

#### 2.2.2. Egfrwt kinase inhibitory assay

EGFR^WT^ kinase inhibiting activities of the target compounds **15–21** were investigated using the homogeneous time resolved fluorescence (HTRF) assay,^44^ with erlotinib as a standard (Table 2). The results revealed that the target compounds exhibited EGFR^WT^ activity with IC_50_ values varying from 0.10 to 3.02 µM. Compounds **15**, **16** and **17** were the most potent against EGFR^WT^ than erlotinib (IC_50_ = 0.32 ± 0.05 μM) with IC_50_ values of 0.22, 0.10 and 0.19 μM, respectively. However, compounds **18** and **20** showed to have similar activities to erlotinib with IC_50_ values of 0.65 and 0.77 µM, respectively. Finally, compounds **19** and **21** exerted moderate activities with IC_50_ values of 3.02 and 2.27 µM, respectively.

**Table 2.** *In vitro* enzymatic inhibitory effects of compounds **15–21** against EGFR^WT^ and EGFR^790M^.

Compounds	EGFR^WT^ IC_50_ (µM)^a^	EGFR^790M^ IC_50_ (µM)^a^
**15**	0.22 ± 0.05	6.89 ± 0.31
**16**	0.10 ± 0.03	4.02 ± 0.19
**17**	0.19 ± 0.09	6.26 ± 0.32
**18**	0.65 ± 0.03	8.16 ± 0.28
**19**	3.02 ± 0.12	14.06 ± 0.51
**20**	0.77 ± 0.05	16.26 ± 0.69
**21**	2.27 ± 0.16	15.26 ± 0.61
**Erlotinib**	0.32 ± 0.05	NT
**Gefitinib**	NT	21.44 ± 0.75

^a^IC_50_ values are the mean ± S.D of three experiments.

NT: Compounds not investigated.

#### 2.2.3. Egfrt790m kinase inhibitory assay

The target compounds **15–21** that showed promising IC_50_ values against EGFR^WT^ were explored further for their inhibiting activities against mutant EGFR^T790M^_._ Gefitinib were investigated as a reference standard. The majority of target compounds inhibited EGFR^T790M^ activity, indicating more potent than gefitinib with IC_50_ values varying from 4.02 to 16.26 µM. In particular, the most potent analogue, compound **16** (IC_50_ = 4.02 ± 0.19 µM), was observed to be 5.27 folds more active than gefitinib (IC_50_ = 21.44 ± 0.75 µM). Compounds **15**, **17**, and **18** were the most potent analogues, with 3.07, 3.38 and 2.59 folds the activity of gefitinib, respectively. Finally compounds **19–21** have inhibitory activities equivalent to gefitinib, with IC_50_ values of 14.06, 16.26, and 15.26 µM, respectively (Table 2).

#### 2.2.4. Correlation of cytotoxicity with EGFR^WT^ inhibition

**Figure 3.**
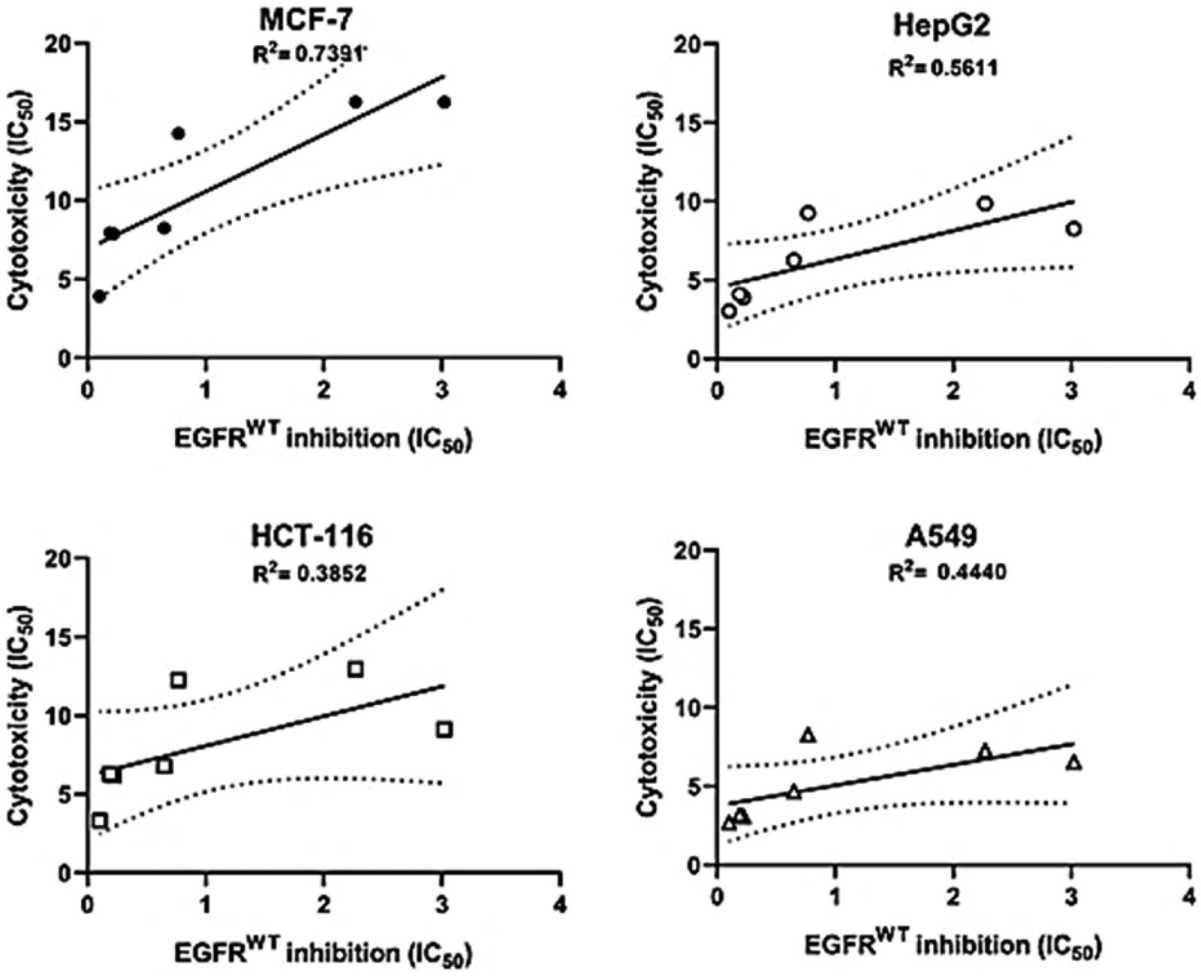
Correlation between EGFR^WT^ inhibition and cytotoxicity on MCF-7, HepG-2, HCT-116 and A549 cell lines.

The target compounds, **15–21** exhibited an inhibitory activity against EGFR^WT^. Next, we evaluated whether the EGFR^WT^ inhibition can lead to an antiproliferative effect in the tested four cell lines. Using the Graph Pad-Prism 5 software, the activity of the tested compounds as EGFR^WT^ inhibitors was plotted against their cytotoxicity in a simple linear regression configuration. The measured coefficients of determination (*R*^2^) represent the relationship between EGFR^WT^ inhibition and the induced antiproliferative activity. The *R*^2^ values for MCF-7, HepG-2, HCT-116 and A549 were 0.7391 (p values: 0.013), 0.5611 (*p* values: 0.05), 0.3852 (*p* values: 0.136) and 0.4440 (*p* values: 0.102), respectively (Figure 3).

#### 2.2.5. Western blotting assay

**Figure 4.**
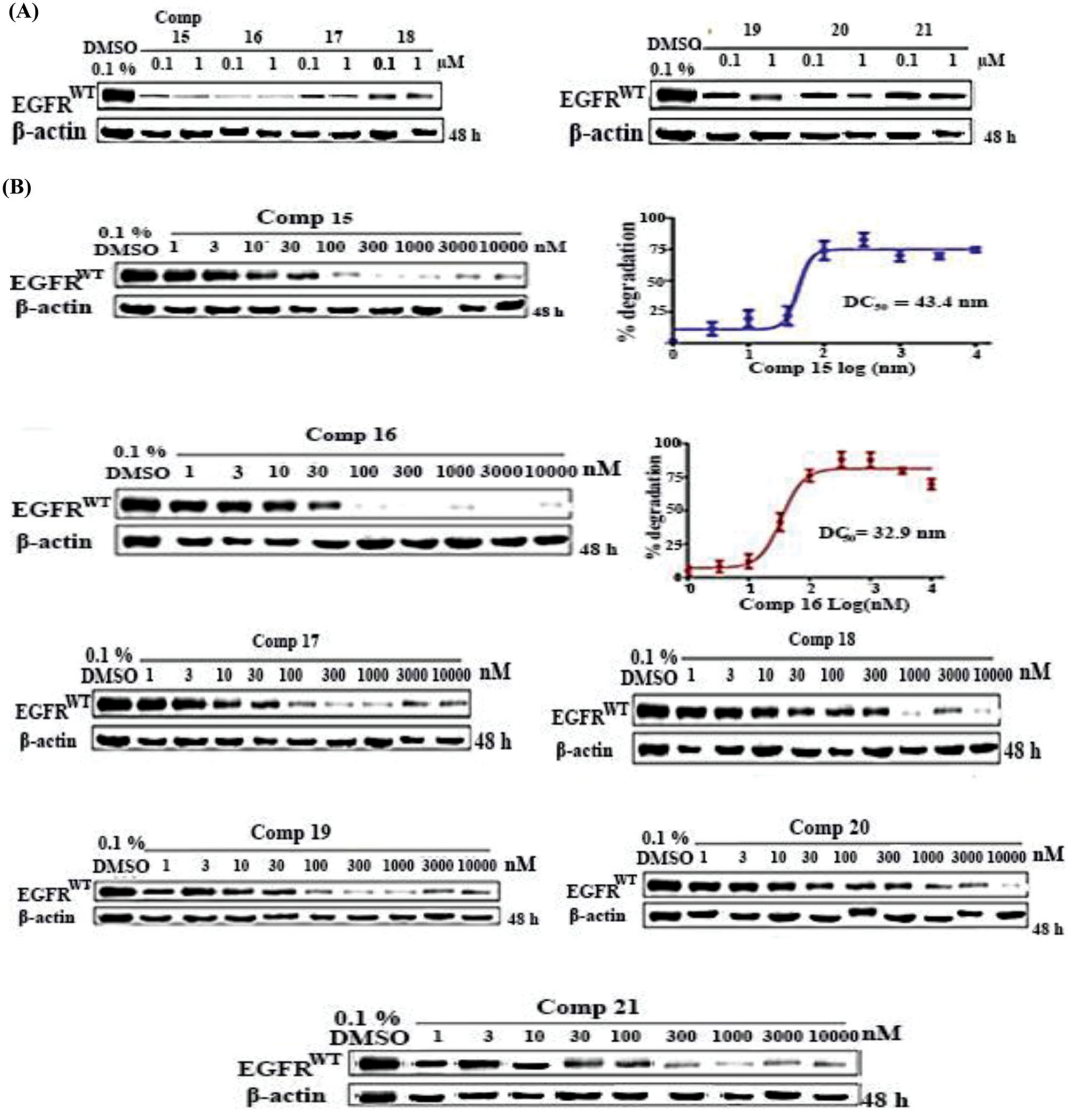
(A) EGFR degradation activities of compounds in A549 cells. Cells were treated for 48 h with concentration of 0.1 and 1 µM. (B) Effects of concentration-dependent EGFR degradation by compounds **15**–**21** in A549 cell lines. Cells were treated for 48 h with a concentration from 1 nM to 10 µM. EGFR protein was examined by Western blotting analysis and EGFR degradation rate was quantified by densitometry and normalised to the corresponding density of β-actin protein (n ¼ 3).

The western blotting analysis showed that, the majority of our target compounds **15–21** are moderate to good degraders (Figure 4(A)). Degradation at the concentration of 1 µM is significantly higher than that at 0.1 µM, indicating a certain concentration-dependent relationship. Following this, the target compounds **15**–**21** tethering various E3 ligases ligand were chosen as representative PROTACs for further degradation study based on temporal data.

We set nine concentration gradients ranging from 1 µM to 10 µM to determine the degradation activity of all tested compounds and calculated the DC_50_ (concentration that caused deletion of 50% of EGFR) values. The results showed that, compounds **15** and **16** can induce EGFR^WT^ degradation in A549 cells in a concentration-dependent manner, with DC_50_ values of 43.4 and 32.9 nM, respectively. At greater concentrations of compound **16**, it exhibited a significant "hook effect" on EGFR degradation, which was caused by the creation of unproductive dimers (rather than productive ternary complex),^35^ while compounds **17–21** were moderately effective at the concentrations of 1 µM to 10 µM (Figure 4(B)).

**Figure 5.**
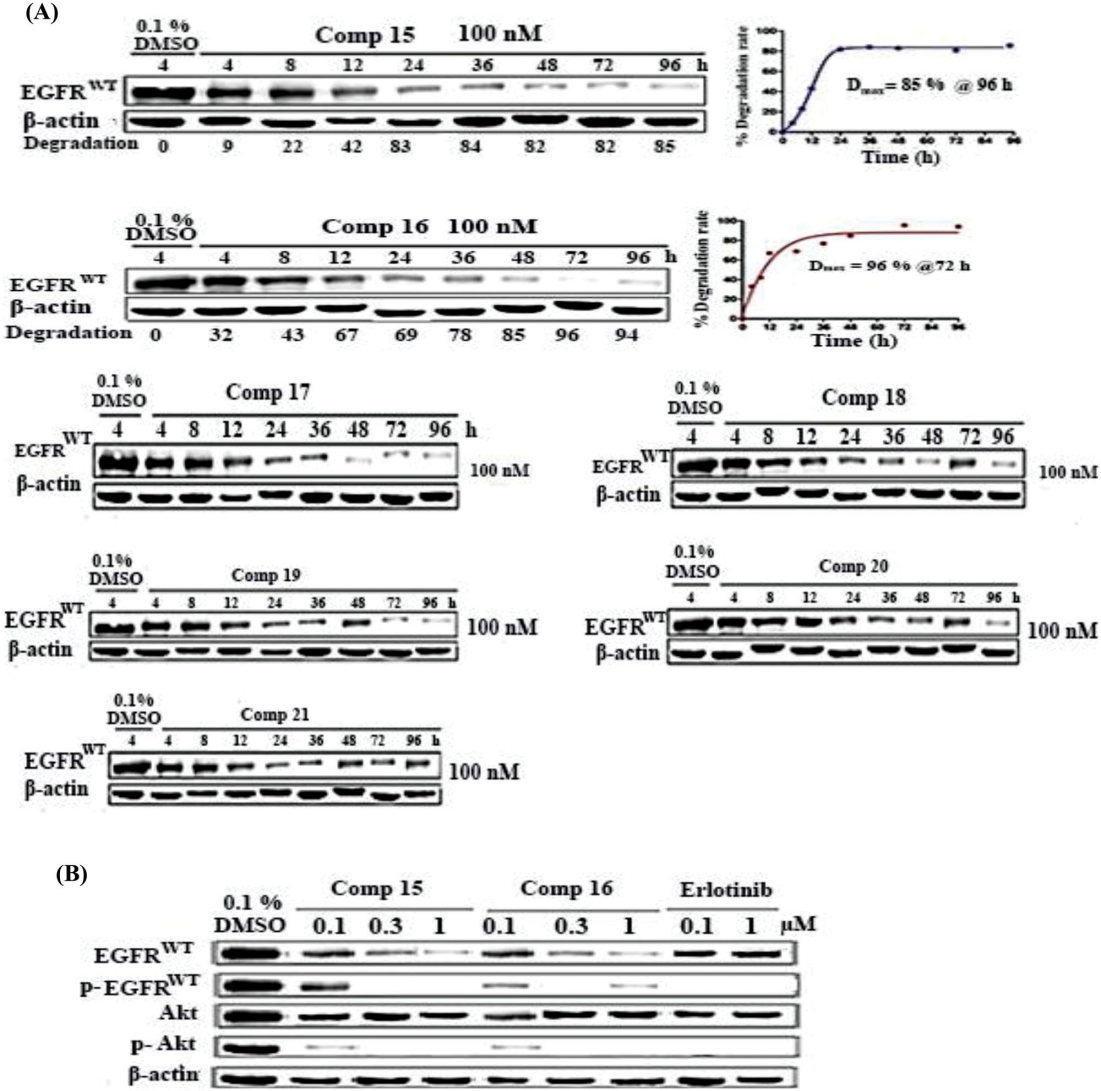
(A) EGFR time-dependent degradation by compound **16** in A549 cell lines. Cells were treated with100 nM of **15–21** for indicated time points. Western blotting was used to evaluate EGFR protein, and densitometry was used to measure EGFR degradation, which was adjusted to the density of β-actin protein (n ¼2). (B) Compounds **15** and **16** have EGFR degradation and phosphorylation inhibitory action in A549 cells. The cells were exposed to concentrations of 0.1, 0.3, and 1 µM. Erlotinib were utilised for comparison.

At a concentration of 100 nM, the time-dependent degradation activities of compounds **15**–**21** were also examined (Figure 5(A)). As the administration duration was extended, the amount of EGFR protein was gradually reduced. At 96 hours, compound **15** reached its maximum degradation rate (D_max_ = 86%) at 96 h, and compound **16** showed the maximum degradation rate (D_max_ = 96%) at 72 h. These results indicate that transmembrane protein degradation is a time-consuming process. Moreover, as shown in (Figure 5(B)), compounds **15** and **16** at the concentrations of 0.1, 0.3 and 1 µM effectively displayed inhibitory activity against EGFR and downstream Akt phosphorylation in a concentration-dependent manner.

Compound **15** inhibited EGFR phosphorylation in a similar way as erlotinib, whereas compounds **15** and **16** have inhibitory actions on Akt phosphorylation that were comparable to erlotinib. In A549 cells, the promising compounds **15** and **16** promoted EGFR degradation with DC_50_ values of 43.4 and 32.9 nM, respectively. Protein-controlling machinery in cells (ubiquitin-proteasome system) UPS was a part of the process.

#### 2.2.6. In vitro *DNA-flow cytometric (cell cycle) analysis*

Cell cycle analysis on MCF-7, HepG-2 and HCT-116 cells was performed for the most potent compound **16**, as indicated by *Wang et al.*^45^ Compound **16** was incubated with MCF-7, HepG-2, and HCT-116 cells for 24 hours at doses equivalent to its IC_50_ against the three cell lines (3.92, 3.02 and 3.32 µM, respectively). Then, the influence of compound **16** on the cell cycle profile was then investigated.

**Figure 6.**
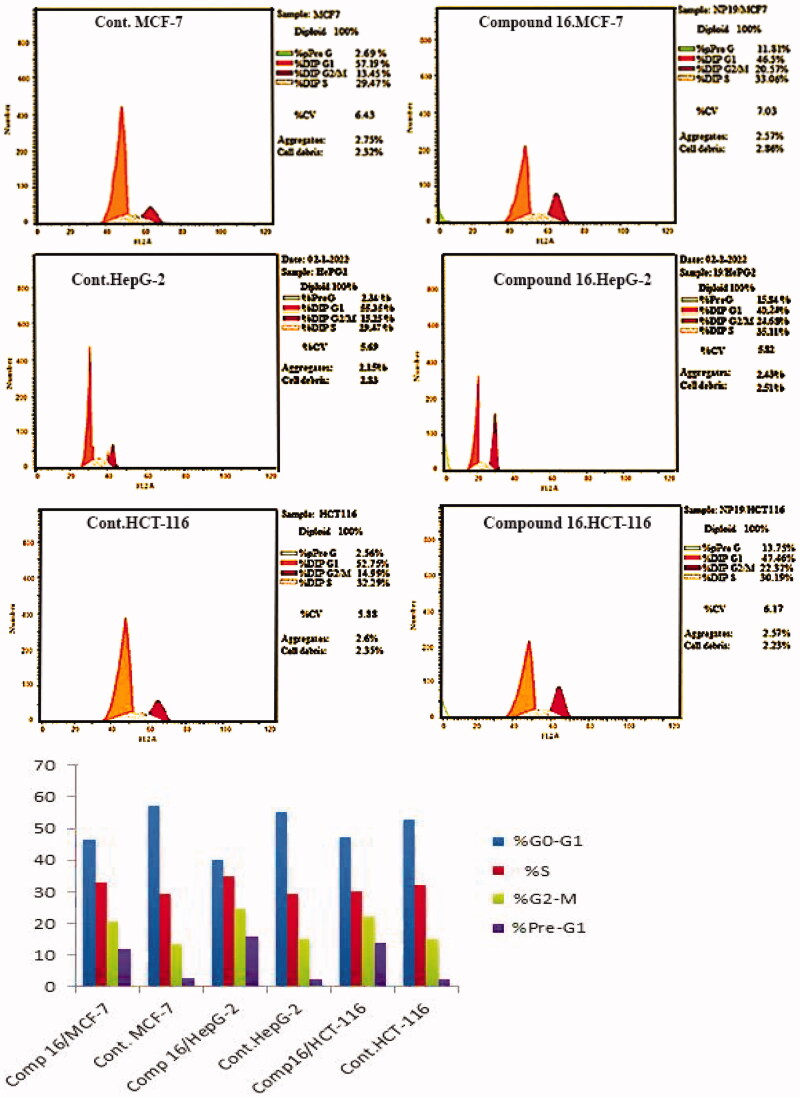
The distribution of MCF-7, HepG-2 and HCT-116 cells after treatment with compound **16**.

When MCF-7 pre-treated to compound **16**, the percentage of cells in pre-G1 and G2-M phases increased by 4.39 and 1.53 fold, respectively, compared to the control. In HepG-2 cells, compound **16** caused 6.76 and 1.61 fold increase in the percentage of cells in pre-G1 and G2-M stages, respectively, as compared to the control. In HCT-116 cells, compound **16** caused 5.37 and 1.49 fold increase in the percentage of cells in pre-G1 and G2-M phases, respectively, as compared to the control. These results clearly showed that compound **16** inhibits the cell cycle in the G2-M phase (Figure 6).

#### 2.2.7. Apoptosis analysis

**Figure 7.**
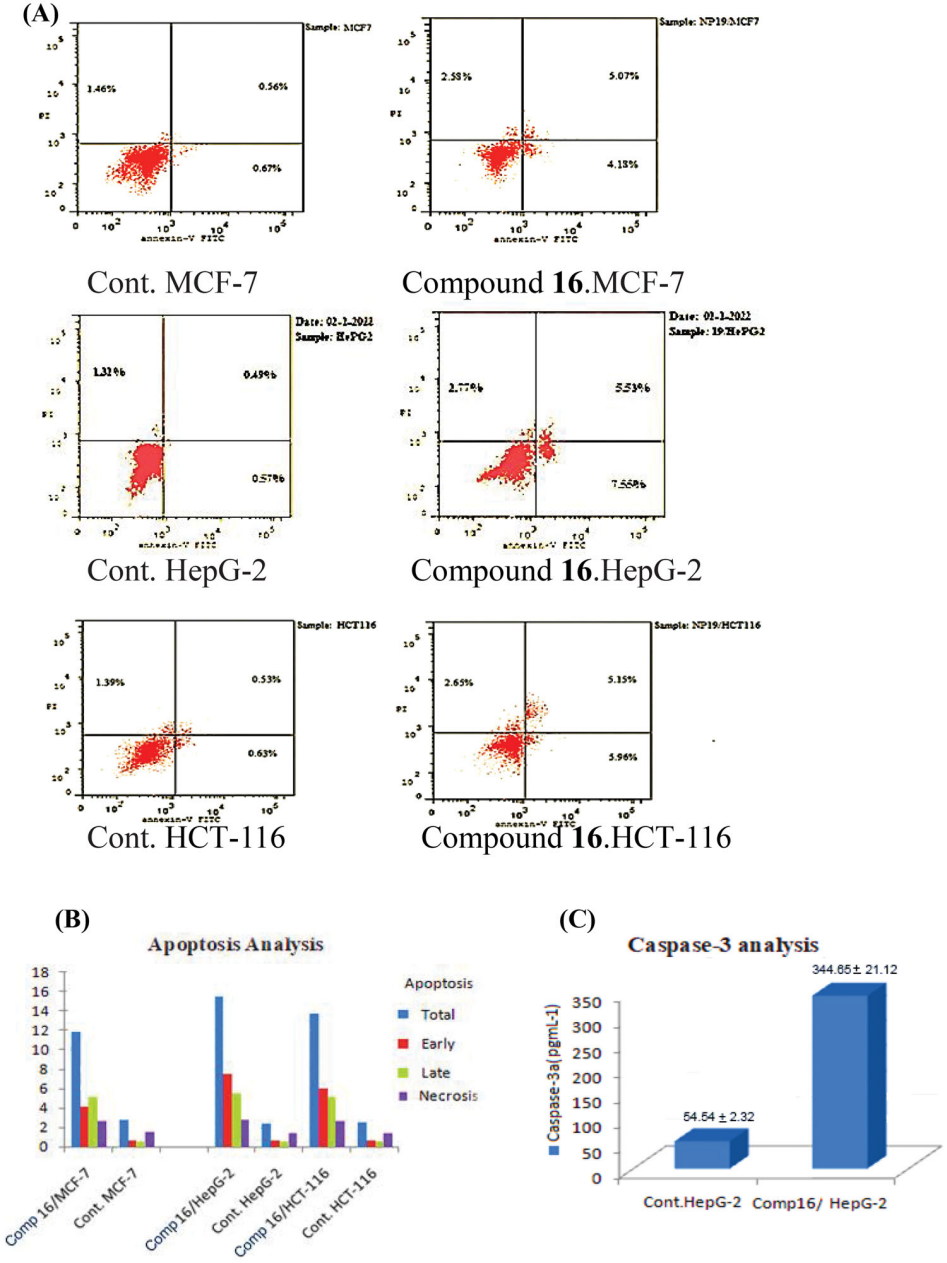
(A) and (B) Apoptosis effect of compound **16** on MCF-7, HepG-2, and HCT-116 cells. (C) Effect compound **16** on caspase-3 levels.

The double staining Annexin V/propidium iodide technique was performed to assess the mechanism of cell death and apoptosis-inducing activity.^46^ For 24 hours, MCF-7, HepG-2, and HCT-116 cells were treated with compound **16** at concentrations of 3.92, 3.02 and 3.32 µM, respectively. As shown in (Figure 7 (A and B).

The results revealed that the application of compound **16** on MCF-7 cells the early apoptosis ratio jumped from 0.67% to 4.18%, and the late apoptosis ratio from 0.56% to 5.07%, compound **16** promoted nearly 9-times for cellular apoptosis, including early and late when compared to control. HepG-2 cells, compound **16** improved the early apoptosis ratio from 0.57% to 7.55%, while the late apoptosis ratio rises from 0.49% to 5.53%. This means that compound **16** caused almost up to 13-folds for both early and late cellular apoptosis upon comparison with the control. Compound **16** enhanced the early apoptosis ratio in HCT-116 cells from 0.63% to 5.96%, as well as the late apoptosis ratio from 0.53% to 5.15%, upon comparison with the control, compound **16** caused nearly 9-times for cellular apoptosis, including early and late. Compound **16** has a significant apoptotic effect against MCF-7, HepG-2, and HCT-116 cells, according to the obtained results.

#### 2.2.8. Caspase-3 determination

The most sensitive cells (HepG-2) were treated with compound **16** at a concentration of 3.02 µM for 24 hours to determine the effect of compound 16 on caspase-3 levels, The results showed that considerable increase the level of caspase-3 (6.31 fold) when compared to control cells (Figure 7 (C)).

### 2.3. In silico studies

#### 2.3.1. Molecular docking

Docking studies were performed for the compounds **15–21** against the ATP binding sites of EGFR-TK Wild-type (EGFR^WT^, PDB:4HJO)^47^ and EGFR-TK mutant type (EGFR^T790M^, PDB: 3W2O).^48^^,^^49^ The docked compounds revealed good binding affinities against EGFR^WT^ (energy score −7.50 to − 8.65 kcal mol^−1^; Table 3).

**Table 3.** The binding free energies of docking the target compounds **15–21** against EGFR**^WT^** and EGFR**^T790M^.**

Compounds	EGFR^WT^ (Kcal mol^–1^)	EGFR^790M^ (Kcal mol^–1^)
**15**	−7.50	−7.95
**16**	−7.38	−7.58
**17**	−7.68	−7.73
**18**	−8.23	−8.13
**19**	−8.65	−8.41
**20**	−8.14	−8.37
**21**	−8.31	−7.97
**Erlotinib**	−8.24	—
**TAK-285**	—	−8.18

**Figure 8.**
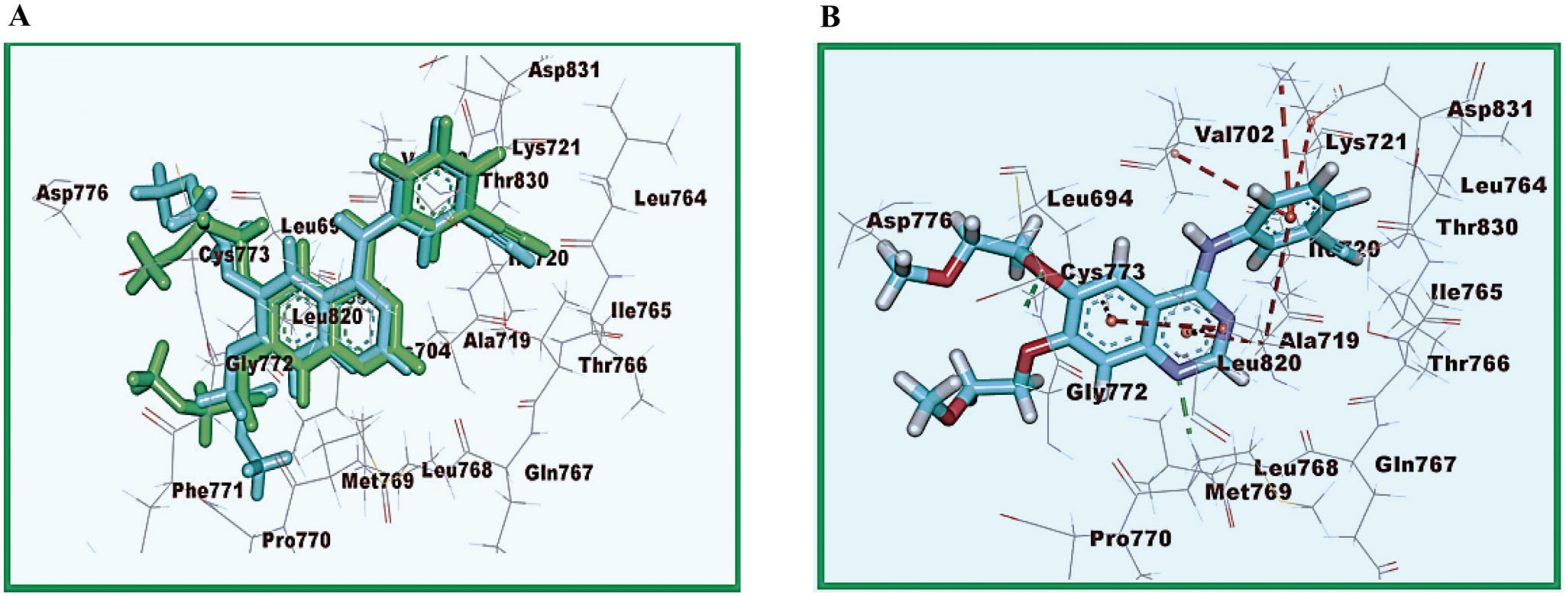
(A) 2D image of the superimposition of the re-docked conformers of erlotinib over the co-crystallized conformers (B) Erlotinib docked into the active site of EGFR^WT^.

The commercial programme Molecular Operating Environment (MOE) 2019.01 was used to construct a molecular docking protocol. The structural coordinates of the co-crystallized inhibitors were used to determine the active binding sites of the target proteins. The docking protocol’s results were verified by re-docking the co-crystallized ligands (erlotinib and TAK-285) inside the active sites of EGFR^WT^ and EGFR^T790M^, respectively. The root mean square deviations (RMSDs) of erlotinib and TAK-285 re-docked conformers and co-crystallized conformers, respectively, were 1.5 and 0.90, demonstrating the docking process’ validity (Figures 8 (A), 10 (A)).

The binding energy of erlotinib as a co-crystallized ligand was −8.24 kcal mol^−1^.The quinazoline moiety been located in the adenine pocket of EGFR^WT^, where the pyrimidine rings creating one hydrogen bonding with Met769 with a distance of 2.22 Å. The quinazoline molecule’s phenyl ring was integrated into *pi*-Sigma linkages with Lue694 and Leu820. The terminal ethynylphenyl moiety was coupled with the hydrophobic pocket I, resulting, hydrophobic interactions with Ala719, Val702 and Lys721 residues. In the hydrophobic region II, two 2-methoxyethoxy groups were discovered generating hydrophobic interactions with Gly772 and Leu694 residues and one hydrogen bond with Cys773 (Figure 8 (B)).

The binding mode of target compound **15** was similar to that of erlotinib. It interacts with the active site through two hydrogen bonds with Asp831 and Lys721 amino acids with a distance of 3.09 and 3.14 Å, respectively and by hydrophobic interactions with Leu694 and Val702 amino acids with bond lengths of 4.32 and 4.60 Å, respectively [binding score = −7.50 kcal mol^−1^] (Supplemental data).

**Figure 9.**
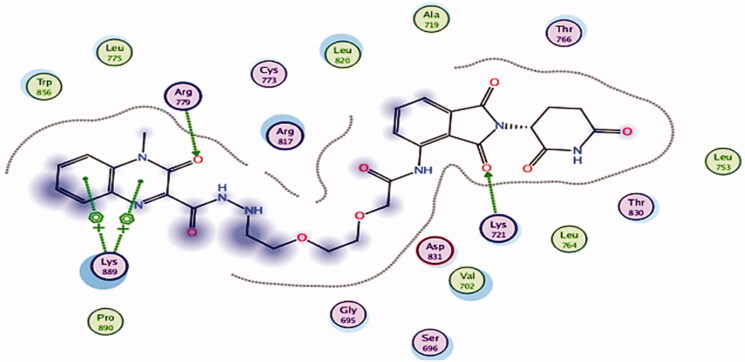
2D diagram representation of compound **16** in the EGFR**^WT^** binding site.

Compound **16** interacted with the active site by two interactions, two hydrogen bonds with Arg779 and Lys721 amino acid with a distance of 3.07 and 2.79 Å, respectively, and by hydrophobic interactions with Lys889 amino acid with a distance of 3.72 and 4.34 Å, respectively [binding score = −7.38 kcal mol^−1^] (Figure 9).

Finally, the interactions between compound **17** and the active site are represented by four hydrogen bonds with Lys851, Lys721 and Phe699 amino acids with a distance of 3.14, 3.14, 2.98 and 3.14 Å respectively [binding score = −7.68 kcal mol^−1^] (Supplemental data).

**Figure 10.**
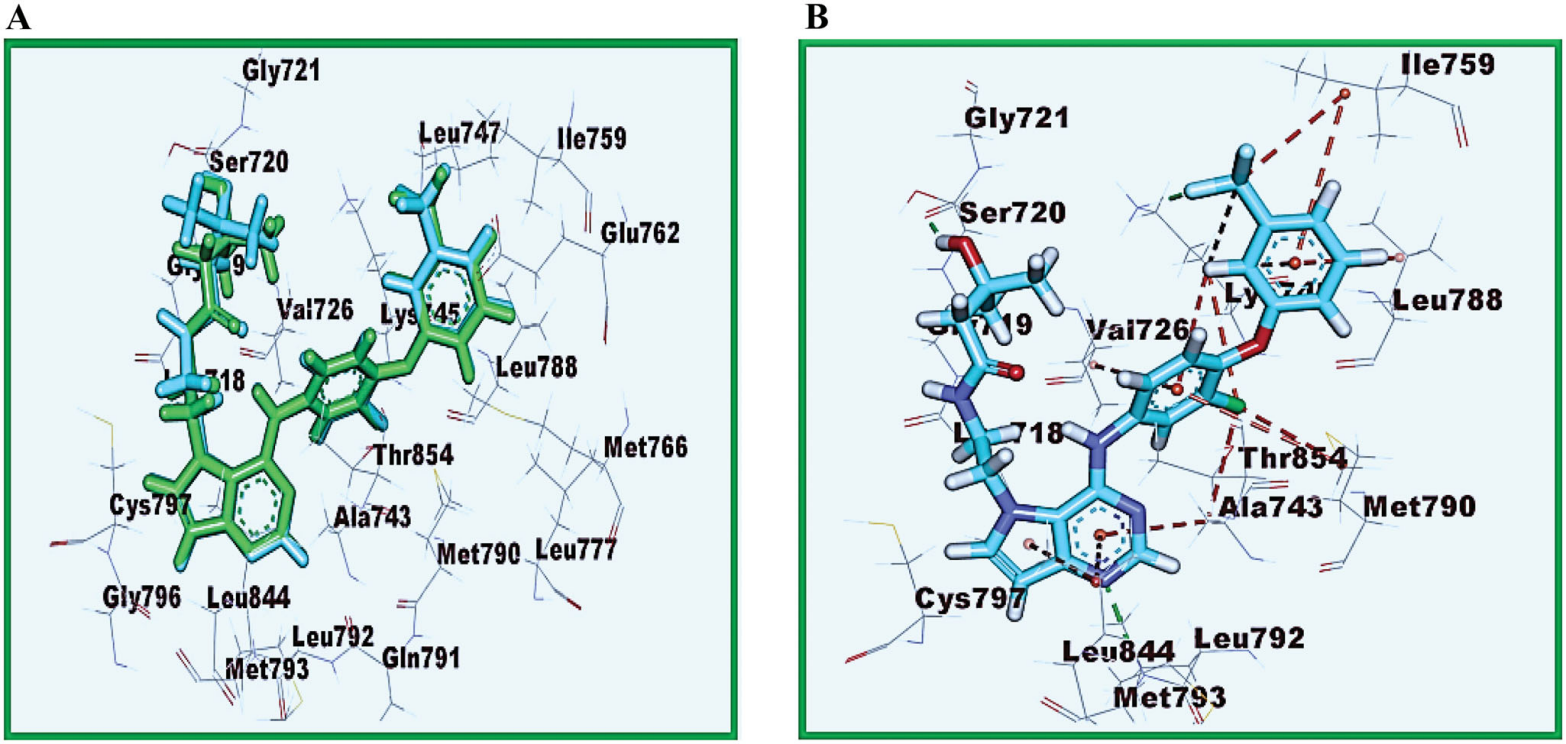
(A) 3D images of the superimposition of the re-docked conformers of TAK-285 over the co-crystallized conformers. (B) Co-crystallized ligand (TAK-285) docked into the active site of EGFR^T790M^.

The docked compounds **15–21** have revealed good binding affinities against EGFR^T790M^ (energy score −7.58 to −8.41 kcal mol^−1^, Table 3). As a co-crystallized ligand, TAK-285’s pyrrolo[3,2-d]pyrimidine moiety occupied the adenine pocket of EGFR^T790M^ (energy score −8.18 kcal mol-1). ALa734 and Leu718 have developed hydrophobic contacts with it. With a bond length of 2.23 Å, the pyrimidine moiety made one hydrogen bond with Met793. The terminal 3-(trifluoromethyl) phenoxy group was integrated into the hydrophobic pocket I, resulting in hydrophobic interactions at Lys745 and Ile759. With a bond length of 1.41 Å, the trifluoromethane group formed one hydrogen bond with Lys745. The N-ethyl-3-hydroxy-3-methylbutanamide moiety occupied the hydrophobic region II, forming one hydrogen bond with Ser720 with a length of 1.79 Å. With Lys745 Val726 and Met790, the central phenyl moiety formed pi-Sigma connections (Figure 10).

Compound **15** bonded with two significant hydrogen bonds with Glu762 and Lys745 amino acids with a distance of 3.11 and 2.84 Å, respectively, and by hydrophobic interactions with Leu718 amino acid with a distance of 4.47 Å [binding score = −7.95 kcal mol^−1^] (Supplemental data).

**Figure 11.**
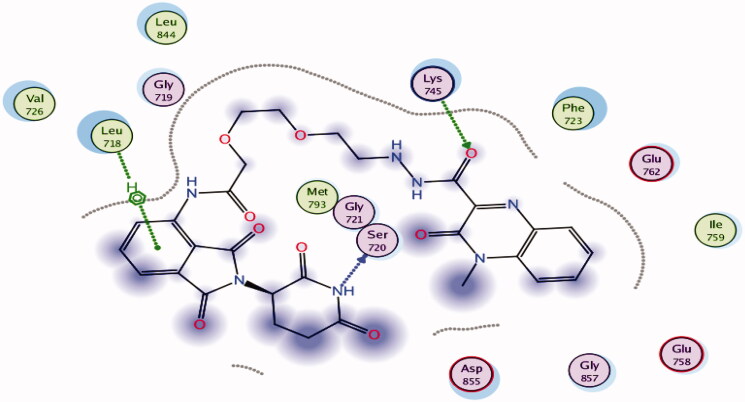
2D diagram representation of compound **16** in the EGFRT^790M^ binding site.

Compound **16** had a binding mechanism that was similar to TAK-285, it interacts with the active site through two hydrogen bonds with Ser720 and Lys745 amino acids with a distance of 3.28 and 3.36 Å, respectively, and by hydrophobic interactions with Leu718 amino acid with bond lengths of 4.54 Å [binding score = −7.58 kcal mol^−1^] (Figure 11).

Compound **17** showed a binding mode identical to TAK-285, it interacts with the active site through four hydrogen bonds with Asp837, Cys797, Leu718, and Met790 amino acids with a distance of 3.48, 3.39, 4.20, and 3.03 Å [binding score = −7.73 kcal mol^−1^] (Supplemental data). The previously mentioned interactions indicate the importance of aromatic moieties, the H-acceptor and H-donor in the designed ligands; this also maintains an appropriate lipophilicity of the designed compound to introduce appropriate pharmacokinetics**.**

## 3. Experimental section

### 3.1. General chemistry

All reagents in this study were provided from Merck, Aldrich, and Fluka. Thin-layer chromatography (TLC) was used to monitor all reactions, with precoated plates of silica gel G/UV-254 of 0.25 mm thickness (Merck 60F254) and UV light (254 nm/365 nm) for visibility. The attenuated total reflection (ATR) method was used to measure infra-red spectra with an FT-IR-ALPHBROKER-Platinum-ATR spectrometer and NMR spectra were performed on Bruker Spectrophotometer (400 MHz, 100 MHz for ^1^H-^13^C NMR, respectively). Chemical shifts are measured in δ values parts per million (ppm) with comparison to the internal reference tetramethylsilane (TMS).

Coupling constants (J) for ^1^H NMR were given in Hertz and expressed as (s) for singlet, (d) for doublet, (t) for triplet, (q) for the quartette, and (m) for multiplet, DMSO-d_6_ was used as a solvent. Microanalyses (C, H, N, and S) and the results were within ± 0.4% of the theoretical values.

Quinoxaline derivatives (**1–3**), isatin hydrazides (**4** and **5**), benzoxazole hydrazide **6**, benzothiazole hydrazide **7** and compounds **8–14** were synthesised according to the reported procedures.^32–36^^,^^44^

#### 3.1.1. General procedure for preparation of compounds 15–21

To NMP (1 ml) solution of key intermediate **14** (0.2 mmol, 1 eq), was added to solution of compounds (**1–7**) (0.2 mmol, 1eq), DIPEA (3eq) was added. The mixture was heating under reflux at 80 °C overnight, mixture was diluted with ethyl acetate before being washed with saturated sodium bicarbonate, water, and brine. The organic layer was dried over sodium sulphate, filtered, and evaporated under reduced pressure, after which it was purified using column chromatography.

##### 3.1.1.1. *N*-(2-(2,6-dioxopiperidin-3-yl)-1,3-dioxoisoindolin-4-yl)-2-(2-(2-(3-oxo-3,9-dihydro-2H-pyrazolo[3,4-*b*]quinoxalin-2-yl)ethoxy)ethoxy)acetamide (15)

White crystals (yield 56%); IR (KBr) ν cm^−1^: 3304-3294 (NH), 3085 (CH aromatic), 2910 (CH aliphatic), 1703 (C = O); ^1^H NMR *δ* ppm; 11.29 (s, IH, NH quinoxalin, exchangeable with D_2_O), 10.72 (br, IH, NH pomalidomide, exchangeable with D_2_O), 10.07 (br, IH, NH piperidine, exchangeable with D_2_O), 8.09–8.07 (d, 2H, *J* = 8.8 Hz, Ar-H), 7.92 (t, 2H, *J* = 7.6 Hz, Ar-H), 7.60 (t, 1H, *J* = 7.6 Hz, Ar-H), 7.45–7.43 (d, 2H, *J* = 8.4 Hz, Ar-H), 5.09 (t, 1H, *J* = 8 Hz, CH), 4.62 (s, 2H, CH_2_), 3.45–3.21 (m, 6H, CH_2_), 2.95 (t, 2H, *J* = 7.8 Hz, CH_2_), 2.60 (t, 2H, *J* = 7.6 Hz, CH_2_), 2.09–2.07 (m, 2H, CH_2_); ^13 ^C NMR *δ* ppm; 179.60, 178.50, 177.80, 177.47, 166.50, 156.68, 154.56, 151.21, 142.30, 139.40, 137.57, 134.40, 131.28, 130.19, 128.98, 128.26, 127.56, 126.58, 124.56, 110.69, 70.81, 69.68, 69.39, 68.18, 56.15, 49.78, 26.50, 24.56; Dept-135 NMR *δ* ppm; 70.81 (exchangeable), 69.68 (exchangeable), 69.39 (exchangeable), 68.18 (exchangeable), 49.78 (exchangeable), 26.50 (exchangeable), 24.56 (exchangeable). Anal. Calcd for C_28_H_25_N_7_O_8_ (587.55): C, 57.24; H, 4.29; N, 16.69; found: C, 57.37; H, 4.18; N, 16.60, %.

##### 3.1.1.2. *N*-(2-(2, 6-dioxopiperidin-3-yl)-1,3-dioxoisoindolin-4-yl)-2-(2-(2-(2-(4-methyl-3-oxo-3, 4-dihydroquinoxaline-2-carbonyl) hydrazinyl)ethoxy)ethoxy) acetamide (16)

White crystals (yield 60%); IR (KBr) ν cm^−1^: 3305-3272 (NH), 3035 (CH aromatic), 2889 (CH aliphatic), 1699 (C = O); ^1^H NMR *δ* ppm; 11.07 (br, IH, NH, CONHNH, exchangeable with D_2_O), 10.60 (br, IH, NH piperidine, exchangeable with D_2_O), 10.09 (s, IH, NH pomalidomide, exchangeable with D_2_O), 7.97–7.95 (d, 2H, *J* = 8.4 Hz, Ar-H), 7.93 (t, 2H, *J* = 7.6 Hz, Ar-H), 7.58 (t, 1H, *J* = 7.6 Hz, Ar-H), 7.35–7.33 (d, 2H, *J* = 8.4 Hz, Ar-H), 6.21 (br, IH, NH, CONHNH, exchangeable with D_2_O), 4.92 (t, 1H, *J* = 7.2 Hz, CH), 4.59 (s, 2H, CH_2_), 3.90–3.88 (m, 6H, CH_2_), 3.45 (s, 3H, CH_3_), 2.95 (t, 2H, *J* = 7.4 Hz, CH_2_), 2.60 (t, 2H, *J* = 7.6 Hz, CH_2_), 235–2.33 (m, 2H, CH_2_); ^13 ^C NMR *δ* ppm; 168.52, 168.14, 167.80,167.21, 162.76, 153.47, 152.21, 150.87, 142.76, 138.25, 136.67, 134.67, 131.21, 130.07, 128.76, 128.13, 127.47, 125.18, 123.47, 111.21, 68.81, 68.05, 67.68, 67.07, 58.21, 47.80, 28.45, 27.80, 23.47; Dept-135 NMR *δ* ppm; 68.81 (exchangeable), 68.05 (exchangeable), 67.68 (exchangeable), 67.07 (exchangeable), 47.80 (exchangeable), 27.80 (exchangeable), 23.47 (exchangeable); Anal. Calcd for C_29_H_29_N_7_O_9_ (619.59): C, 56.22; H, 4.72; N, 15.82; found: C, 56.30; H, 4.59; N, 15.90, %.

##### 3.1.1.3. *N*-(2-(2-(2-(2-(2-(2,6-dioxopiperidin-3-yl)-1,3-dioxoisoindolin-4-ylamino)-2-oxoethoxy)ethoxy)ethoxy)ethyl)-3-oxo-3,4-dihydroquinoxaline-2-carboxamide (17)

White crystals (yield 58%); IR (KBr) ν cm^−1^: 3335-3299 (NH), 3027 (CH aromatic), 2889 (CH aliphatic), 1695 (C = O); ^1^H NMR *δ* ppm; 11.11 (br, IH, NH quinoxaline, exchangeable with D_2_O), 10.95 (br, IH, NH piperidine, exchangeable with D_2_O), 10.45 (s, IH, NH pomalidomide, exchangeable with D_2_O), 7.98–7.96 (d, 2H, *J* = 8.4 Hz, Ar-H), 7.86 (t, 2H, *J* = 7.6 Hz, Ar-H), 7.57 (t, 1H, *J* = 7.6 Hz, Ar-H), 7.48–7.46 (d, 2H, *J* = 8.4 Hz, Ar-H), 6.83 (s, IH, NH, NHCH_2_, exchangeable with D_2_O), 4.95 (t, 1H, *J* = 7.2 Hz, CH), 4.57 (s, 2H, CH_2_), 4.15 (t, 2H, *J* = 7.6 Hz, CH_2_), 3.85–3.83 (m, 6H, CH_2_), 3.58 (t, 2H, *J* = 7.6 Hz, CH_2_), 2.85–2.83 (m, 2H, CH_2_), 2.48 (t, 2H, *J* = 8 Hz, CH_2_), 2.15 (t, 2H, *J* = 8 Hz, CH_2_); ^13 ^C NMR *δ* ppm; 178.63, 177.28, 176.51, 175.45, 164.62, 157.77, 156.24, 150.87, 144.56, 139.54, 137.82, 134.67, 131.41, 130.38, 129.17, 127.98, 127.19, 126.98, 123.47, 114.56, 79.60, 73.47, 69.98, 69.19, 67.80, 60.60, 54.56, 27.19, 26.98, 23.47; Dept-135 NMR *δ* ppm; 79.60 (exchangeable), 73.47 (exchangeable), 69.98 (exchangeable), 69.19 (exchangeable), 67.80 (exchangeable), 54.56 (exchangeable), 27.19 (exchangeable), 26.98 (exchangeable), 23.47 (exchangeable); Anal. Calcd for C_30_H_30_N_6_O_10_ (634.60): C, 56.78; H, 4.77; N, 13.24; found: C, 56.63; H, 4.63; N, 13.32, %.

##### 3.1.1.4. *N*-(2-(2,6-dioxopiperidin-3-yl)-1,3-dioxoisoindolin-4-yl)-2-(2-(2-(2-(4-((2-oxoindolin-3-ylidene)amino)benzoyl)hydrazineyl)ethoxy)ethoxy)acetamide (18)

Yellowish white crystals (yield 52%); IR (KBr) ν cm^−1^: 3297-3288 (NH), 3025 (CH aromatic), 2881 (CH aliphatic), 1693 (C = O); ^1^H NMR *δ* ppm; 11.11 (s, IH, NH isatin, exchangeable with D_2_O), 10.76 (s, IH, NH piperidine, exchangeable with D_2_O), 10.15 (br, IH, NH pomalidomide, exchangeable with D_2_O), 9.65 (s, IH, NH, CONHNH, exchangeable with D_2_O), 7.98–7.96 (d, 4H, *J* = 8 Hz, Ar-H_._), 7.90 (t, 4H, *J* = 7.8 Hz, Ar-H), 7.68 (t, 1H, *J* = 7.8 Hz, Ar-H), 7.50–7.48 (d, 2H, *J* = 8 Hz, Ar-H), 6.43 (s, IH, NH, CONHNH, exchangeable with D_2_O), 4.95 (t, 1H, *J* = 7.8 Hz, CH), 4.29 (s, 2H, CH_2_), 3.88 (t, 4H, *J* = 7.6 Hz, CH_2_), 3.67 (t, 4H, *J* = 7.8 Hz, CH_2_), 3.06 (t, 2H, *J* = 7.6 Hz, CH_2_), 2.57–2.55 (m, 2H, CH_2_); ^13 ^C NMR *δ* ppm; 174.56, 169.98, 169.19, 167.80, 167.19, 166.80, 164.50, 157.50, 156.56, 154.56, 150.50, 144.56, 140.81, 139.56, 137.50, 131.40, 130.19, 129.50, 126.50, 124.56, 123.47, 119.80, 119.19, 117.80, 114.60, 113.47, 69.98, 69.60, 68.40, 67.18, 64.40, 54.81, 29.78, 21.40; Dept-135 NMR *δ* ppm; 69.98 (exchangeable), 69.60 (exchangeable), 68.40 (exchangeable), 67.18 (exchangeable), 54.81 (exchangeable), 29.78 (exchangeable), 21.40 (exchangeable); Anal. Calcd for C_34_H_31_N_7_O_9_ (681.66): C, 59.91; H, 4.58; N, 14.38; found: C, 60.03; H, 4.45; N, 14.29, %.

##### 3.1.1.5. *N*-(2-(2,6-dioxopiperidin-3-yl)-1,3-dioxoisoindolin-4-yl)-2-(2-(2-(2-(4-(1-methyl-2-oxoindolin-3-ylideneamino)benzoyl)hydrazinyl)ethoxy)ethoxy)acetamide (19)

Yellowish white crystals (yield 56%); IR (KBr) ν cm^−1^: 3315-3294 (NH), 3022 (CH aromatic), 2921 (CH aliphatic), 1705 (C = O); ^1^H NMR *δ* ppm; 11.07 (s, IH, NH piperidine exchangeable with D_2_O), 10.41 (br, IH, NH pomalidomide, exchangeable with D_2_O), 9.52 (s, IH, NH, CONHNH, exchangeable with D_2_O), 8.09–8.07 (d, 4H, *J =* 8.4 Hz, Ar-H_._), 7.96 (t, 4H, *J =* 7.6 Hz, Ar-H), 7.76 (t, 1H, *J =* 7.6 Hz, Ar-H), 7.43–7.41 (d, 2H, *J* = 8.4 Hz, Ar-H), 6.29 (s, IH, NH, CONHNH, exchangeable with D_2_O), 4.86 (t, 1H, *J =* 7.8 Hz, CH), 4.59 (s, 2H, CH_2_), 3.96 (t, 4H, *J* = 7.6 Hz, CH_2_), 3.74 (t, 4H, *J* = 7.8 Hz, CH_2_), 3.51 (s, 3H, CH_3_), 2.96 (t, 2H, *J* = 7.6 Hz, CH_2_), 258-2.56 (m, 2H, CH_2_); ^13 ^C NMR *δ* ppm; 171.40, 170.50, 169.98, 167.98, 166.50, 164.60, 163.47, 159.98, 157.98, 156.50, 151.40, 144.60, 143.47, 139.98, 137.98, 131.50, 130.60, 129.47, 127.60, 126.50, 123.47, 119.98, 119.50, 117.60, 116.50, 114.60, 69.80, 69.60, 68.47, 67.18, 63.47, 54.56, 26.47, 24.60, 23.40; Dept-135 NMR *δ* ppm; 69.80 (exchangeable), 69.60 (exchangeable), 68.47 (exchangeable), 67.18 (exchangeable), 54.56 (exchangeable), 24.60 (exchangeable), 23.40 (exchangeable); Anal. Calcd for C_35_H_33_N_7_O_9_ (695.69): C, 60.43; H, 4.78; N, 14.09; found: C, 60.29; H, 4.94; N, 14.17, %.

##### 3.1.1.6. 2-(2-(2-(2-(2-(benzo[d]oxazol-2-ylthio)acetyl)hydrazinyl)ethoxy)ethoxy)-*N*-(2-(2,6-dioxopiperidin-3-yl)-1, 3-dioxoisoindolin-4-yl)acetamide (20)

White crystals (yield 50%); IR (KBr) ν cm^−1^: 3337-3289 (NH), 3031 (CH aromatic), 2859 (CH aliphatic), 1691 (C = O); ^1^H NMR *δ* ppm; 10.76 (s, IH, NH piperidine, exchangeable with D_2_O), 10.15 (br, IH, NH pomalidomide, exchangeable with D_2_O), 9.57 (s, IH, NH, CONHNH, exchangeable with D_2_O), 8.07–8.05 (d, 2H, *J* = 8.4 Hz, Ar-H), 7.97 (t, 2H, *J* = 7.8 Hz, Ar-H), 7.79 (t, 1H, *J* = 7.8 Hz, Ar-H), 7.57-7.55 (d, 2H, *J* = 8.4 Hz, Ar-H), 6.76 (s, IH, NH, CONHNH, exchangeable with D_2_O), 4.55 (t, 1H, *J* = 8.4 Hz, CH), 4.15 (s, 2H, CH_2_), 3.92 (s, 2H, CH_2_), 3.77-3.75 (m, 6H, CH_2_), 3.56 (t, 2H, *J* = 7.8 Hz, CH_2_), 2.77- 2.75 (m, 2H, CH_2_), 2.57 (t, 2H, *J* = 8.6, CH_2_); ^13 ^C NMR *δ* ppm; 174.40, 171.80, 170.50, 169.60, 169.19, 168.18, 166.50, 152.80, 142.80, 140.60, 138.18, 136.50, 127.80, 126.50, 124.80, 124.40, 119.70, 117.80, 112.80, 69.60, 69.19, 68.18, 66.50, 63.47, 52.80, 42.80, 29.60, 28.18; Dept-135 NMR *δ* ppm; 69.60 (exchangeable), 69.19 (exchangeable), 68.18 (exchangeable), 66.50 (exchangeable), 52.80 (exchangeable), 42.80 (exchangeable), 29.60 (exchangeable), 28.18 (exchangeable); Anal. Calcd for C_28_H_28_N_6_O_9_S (624.63): C, 53.84; H, 4.52; N, 13.45; S, 5.13; found: C, 53.95; H, 4.38; N, 13.38, S, 5.03%.

##### 3.1.1.7. 2-(2-(2-(2-(2-(benzo[d]thiazol-2-ylthio)acetyl)hydrazinyl)ethoxy)ethoxy)-*N*-(2-(2, 6-dioxopiperidin-3-yl)-1,3-dioxoisoindolin-4-yl)acetamide (21)

White crystals (yield 52%); IR (KBr) ν cm^−1^: 3299-3287 (NH), 3029 (CH aromatic), 2899 (CH aliphatic), 1690 (C = O); ^1^H NMR *δ* ppm; 11.22 (s, IH, NH piperidine, exchangeable with D_2_O), 10.92 (s, IH, NH pomalidomide, exchangeable with D_2_O), 10.33 (br, IH, NH, CONHNH, exchangeable with D_2_O), 8.09-8.07 (d, 2H, *J* = 8.4 Hz, Ar-H), 7.97 (t, 2H, *J* = 7.6 Hz, Ar-H), 7.79 (t, 1H, *J* = 7.6 Hz, Ar-H), 7.57- 7.55 (d, 2H, *J* = 8.4 Hz, Ar-H), 6.88 (s, IH, NH, CONHNH, exchangeable with D_2_O), 4.57 (t, 1H, *J* = 7.6 Hz, CH), 4.12 (s, 2H, CH_2_), 3.95 (s, 2H, CH_2_), 3.77-3.75 (m, 6H, CH_2_), 3.57 (t, 2H, *J* = 7.6 Hz, CH_2_), 2.79 (t, 2H, *J* = 7.6, CH_2_), 2.57-2.55 (m, 2H, CH_2_); ^13 ^C NMR *δ* ppm; 172.80, 171.80, 170.69, 169.88, 169.09, 167.80, 167.09, 154.88, 142.80, 141.60, 139.88, 139.09, 127.88, 127.09, 124.88, 124.09, 119.60, 118.18, 114.80, 69.88, 69.09, 68.18, 67.80, 64.80, 57.80, 50.78, 24.80, 21.40; Dept-135 NMR *δ* ppm; 69.88 (exchangeable), 69.09 (exchangeable), 68.18 (exchangeable), 67.80 (exchangeable), 57.80 (exchangeable), 50.78 (exchangeable), 24.80 (exchangeable), 21.40 (exchangeable); Anal. Calcd for C_28_H_28_N_6_O_8_S_2_ (640.69): C, 52.49; H, 4.41; N, 13.12; S, 10.01; found: C, 52.60; H, 4.29; N, 13.04, S, 9.95%.

### 3.2. Biological evaluation

#### 3.2.1. In vitro *antiproliferative activities*

Using the MTT assay technique, the antiproliferative properties of target compounds **15–21** were evaluated *in vitro* against MCF-7, HepG-2, HCT-116, and A549 cell lines.^39^^,^^40^^,^^50^^,^^51^ The used cell lines were procured from ATCC (American Type Culture Collection). The anti-proliferative activity was quantified in the following way. At a density of 3–8 × 10^3^ cells per well, human cancer cell lines were introduced to 96-well plates. The wells were then incubated at 37 °C for 12 hours in 5% CO_2_ incubator. To determine the DMSO content, the culture media was swapped with 0.1 ml of fresh medium containing graded amounts of the test compounds for each well. Incubation time for the wells was two days. The cells were then cultured in 100 µl MTT solution (5 µg ml^−1^) for another 4 hours in each well.

The absorbance of each well was measured at 490 nm with an automated ELISA reader system (TECAN, CHE) after MTT-formazan crystals were dissolved in 100 µl of DMSO. The IC_50_ values were calculated using nonlinear regression fitting models (Graph Pad, Prism Version 5). The results were expressed as means ± SD and were based on the average of three separate, duplicate trials.

#### 3.2.2. Egfrwt and EGFR^T790M^ kinase inhibitory assay

The inhibitory actions of target compounds **15–21** against both EGFR^WT^ and EGFR^T790M^ were investigated further after they demonstrated promising IC_50_ values against target cell lines. This test used HTRF^44^ assay with EGFR^WT^ and EGFR^T790M^ (Sigma). For the first 5 minutes, the evaluated compounds were incubated with EGFR^WT^ and/or EGFR^T790M^ and their substrates in the enzymatic buffer. ATP (1.65 µM) was allowed to react to start the enzymatic activity. The assay was performed at 37 °C for 30 minutes. The addition of EDTA-containing detection reagents halted the process. After one hour detection period, GraphPad Prism 5.0 was used to calculate the IC_50_ values. For each concentration, three separate trials were carried out.

#### 3.2.3. Western bolt assay

Cells were seeded in 6-well plates at 1 × 10^6^ per well, incubated at 37 °C with 5% CO_2_ for 24 h before drug exposure. Cells were treated with different concentrations of synthesised compounds for 96 h, then collected and suspended in lysis buffer (Beyotime) and centrifuged for 20 min at 12000 rpm, later removed the insoluble material. The same amounts of proteins were loaded and separated by 8% sodium dodecyl sulphate-polyacrylamide gel electrophoresis (SDS-PAGE) and transferred to polyvinylidene fluoride membranes (Millipore) after that. The results were detected by an enhanced chemiluminescence system (Mill-pore). The anti-EGFR was diluted at1:1000 (Cell Signalling Technology) were diluted at 1:2000. Anti-actin and the secondary antibodies (Xi’an Zhuangzhi Biotechnology Co., Ltd.) were diluted at 1:5000 and 1:10000, respectively.

#### 3.2.4. Cell cycle analysis

MCF-7, HepG-2, and HCT-116 cells were given the most active compound **16** at concentrations of 3.92, 3.02 and 3.32 µM for 24 hours, respectively. The cells that had been tested were then trypsinized and washed in sterilised phosphate buffer saline (PBS). The collected cells were fixed with cold ethanol (100%, 1.5 ml). According to the manufacturer’s instructions, a Cycle TESTTM PLUS DNA Reagent Kit was used to dye the cells (BD Biosciences, San Jose, CA). A flow cytometer was used to assess cell-cycle distribution.^52^

#### 3.2.5. Cell apoptosis assay

MCF-7, HepG-2, and HCT-116 cells were seeded and grown overnight, then treated for 24 hours with compound **16** at concentrations of 3.92, 3.02, and 3.32 µM, respectively, to test if it caused apoptosis. The negative control was chosen to be DMSO. The cells were then collected and washed twice in PBS. The cells were separated using centrifugation. An apoptosis detection kit (BD Biosciences, SanJose, CA) was used in this investigation. Following the manufacturer’s instructions, the cells were dyed with Annexin V-FITC and propidium iodide (PI) in the binding buffer for 20 minutes at 37 °C in the darkness. A flow cytometer was used to assess the binding of Annexin V-FITC and PI. The frequencies in all quadrants were investigated using the Flowjo program.^53^

#### 3.2.6. Caspase-3 determination

Caspase-3 activation was measured using a Caspase-3 ELISA Kit (KHO1091).

### 3.3. In silico studies

#### 3.3.1. Docking studies

From the Protein Data Bank (PDB) (http://www.pdb.org), the crystal structures of the target enzymes EGFR^WT^ (PDB ID: 4HJO) and EGFR^T790M^ (PDB ID: 3W2O) were downloaded. The docking analysis was performed using the Molecular Operating Environment (MOE) using the reported procedure^45^^,^^47^ as described in Supplementary Data.

## 4. Conclusions

We have developed a new class of EGFR PROTACs degraders which were based on pomalidomide. All compounds were evaluated for antiproliferative effects *in vitro* and demonstrated potent inhibitory effect on MCF-7, HepG-2, HCT-116, and A549 cell lines. Compound **16** showed to be 5.55, 4.34, 5.04, and 7.18 folds more active than erlotinib in MCF-7, HepG-2, HCT-116, and A549 cells, respectively. For the target compounds **15–21**, inhibitory properties against two isoforms, EGFR^WT^ and EGFR^T790M^, were examined. The target compounds showed promising activities towards both wild-type and mutant forms. Compound **16** revealed to be the most effective EGFR inhibitor with IC_50_ values of 0.10 and 4.02 µM against EGFR^WT^ and EGFR^T790M^, respectively. To have a better understanding of the effect of the target compounds on cancer cell growth inhibition, docking experiments were conducted to fully visualise and interpret compounds’ inhibitory profile against EGFR^WT^ and EGFR^T790M^, and they revealed that the majority of the target compounds have comparable binding mechanisms with the target co-crystallized ligand. Therefore, this study presents compound **16** as a potential promising candidate as an EGFR inhibitor. The western blotting assays analysed the effect of EGFR degradation and the results showed the promising compounds **15** and **16** induced degradation of EGFR in A549 cells with the DC_50_ values of 43.4 and 32.9 nM, respectively. Cellular protein-controlling machinery UPS was involved in this process, compound **16** could effectively degrade EGFR protein through ubiquitination and reached the maximum degradation rate (D_max_ = 96%) at 72 h.

## Supplementary Material

Supplemental Material
