# Supplementary material for: Design, synthesis, and molecular docking studies of novel pomalidomide-based PROTACs as potential anti-cancer agents targeting EGFRWT and EGFRT790M
Source: J Enzyme Inhib Med Chem. 2022 Apr 26;37(1):1196–211. doi: 10.1080/14756366.2022.2062338 (PMC9067978; doi:10.1080/14756366.2022.2062338)
Supplement: Supplemental Material [file IENZ_A_2062338_SM9658.pdf]

Design, Synthesis, and Molecular docking studies of novel pomalidomide-based PROTACs as potential anti-cancer agents targeting EGFR^{WT} and EGFR^{T790M}

Experimental section

3.1.2.1. *N*-(2-(2,6-dioxopiperidin-3-yl)-1,3-dioxoisoindolin-4-yl)-2-(2-(2-(3-oxo-3,9-dihydro-2H-pyrazolo [3, 4-b] quinoxalin-2-yl) ethoxy) ethoxy) acetamide 15.

^{1}H NMR δ ppm

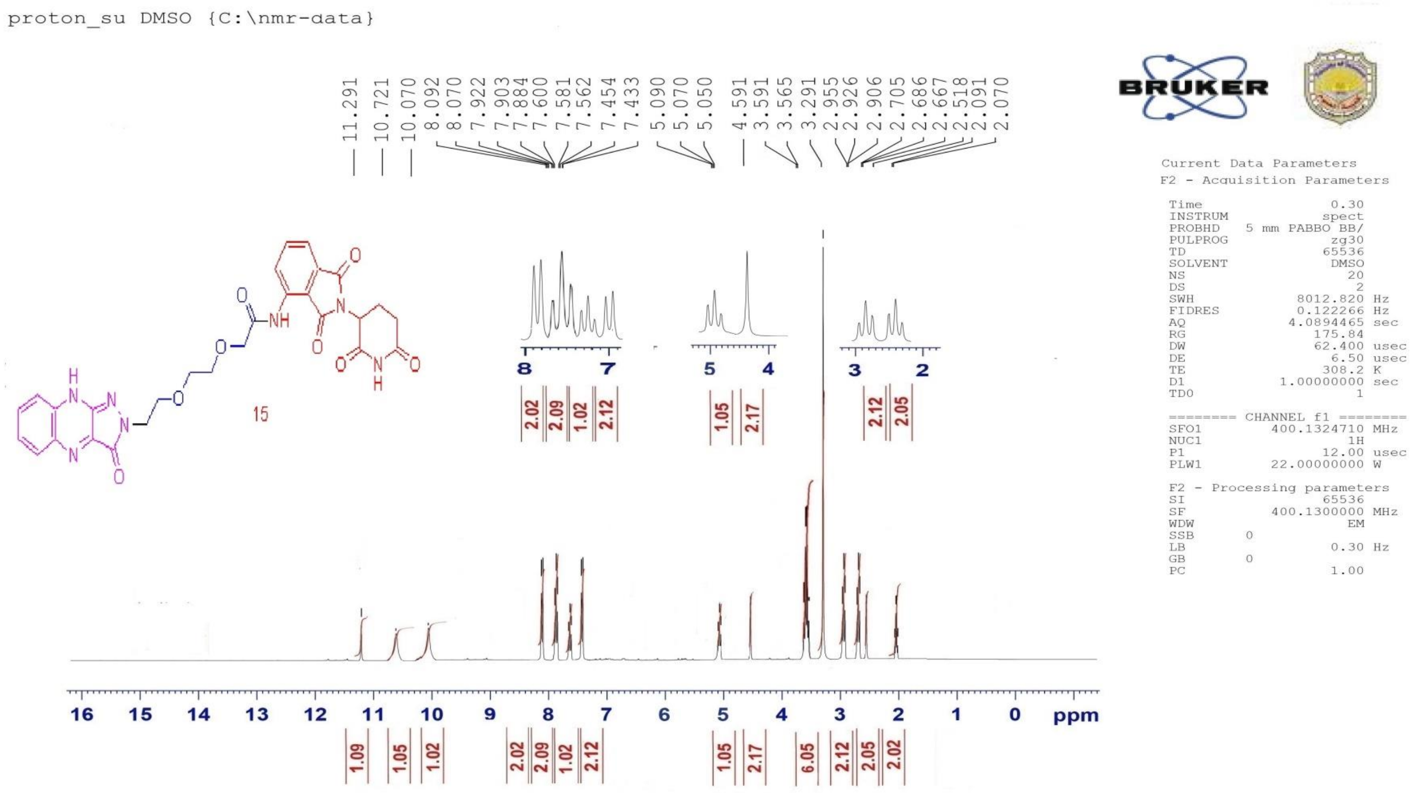

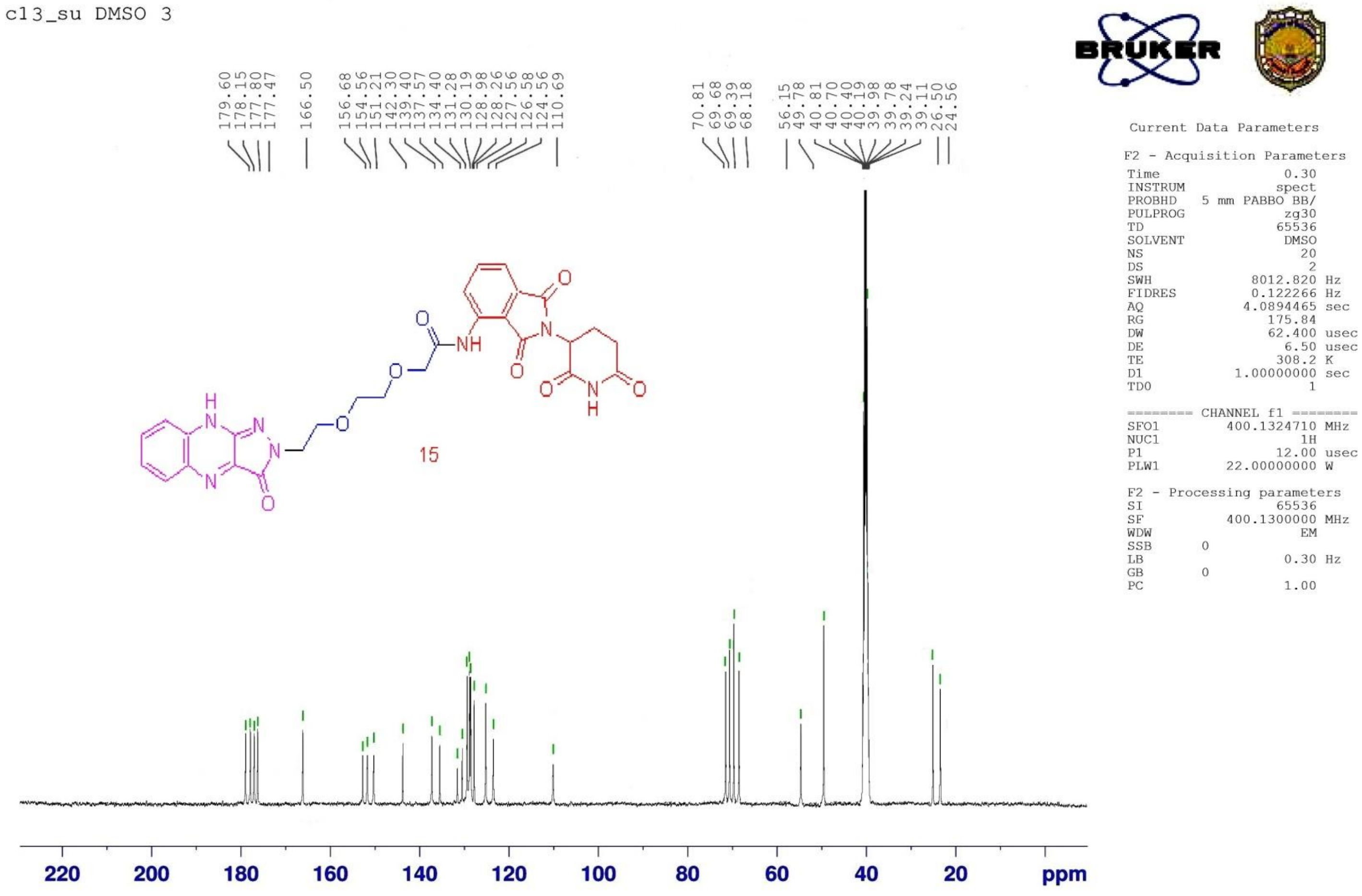

^{13}C NMR δ ppm

IR (KBr) ν cm^{-1}

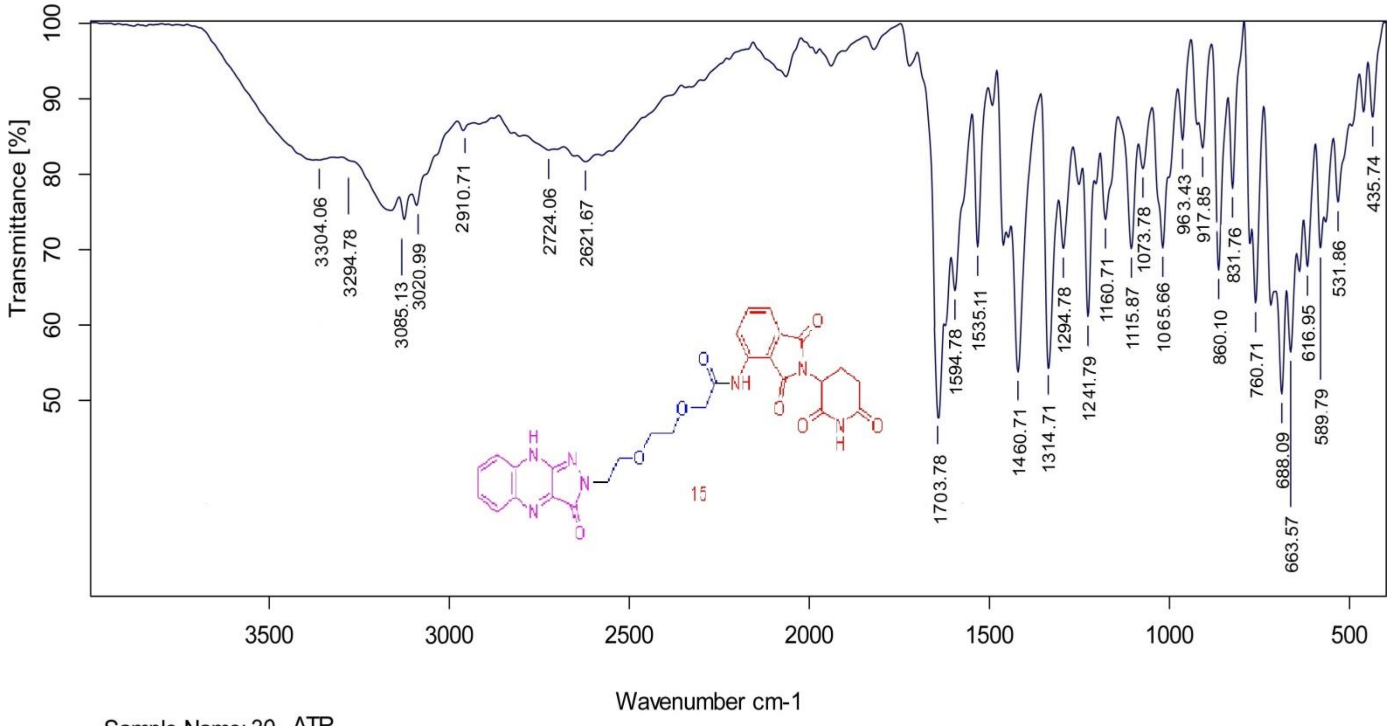

Sample Name: 30 _ATR

3.1.2.2. *N*-(2-(2, 6-dioxopiperidin-3-yl)-1, 3-dioxoisoindolin-4-yl)-2-(2-(2-(2-(4-methyl-3-oxo-3, 4-dihydroquinoxaline-2-carbonyl) hydrazinyl) ethoxy) ethoxy) acetamide 16.

^{1}H NMR δ ppm

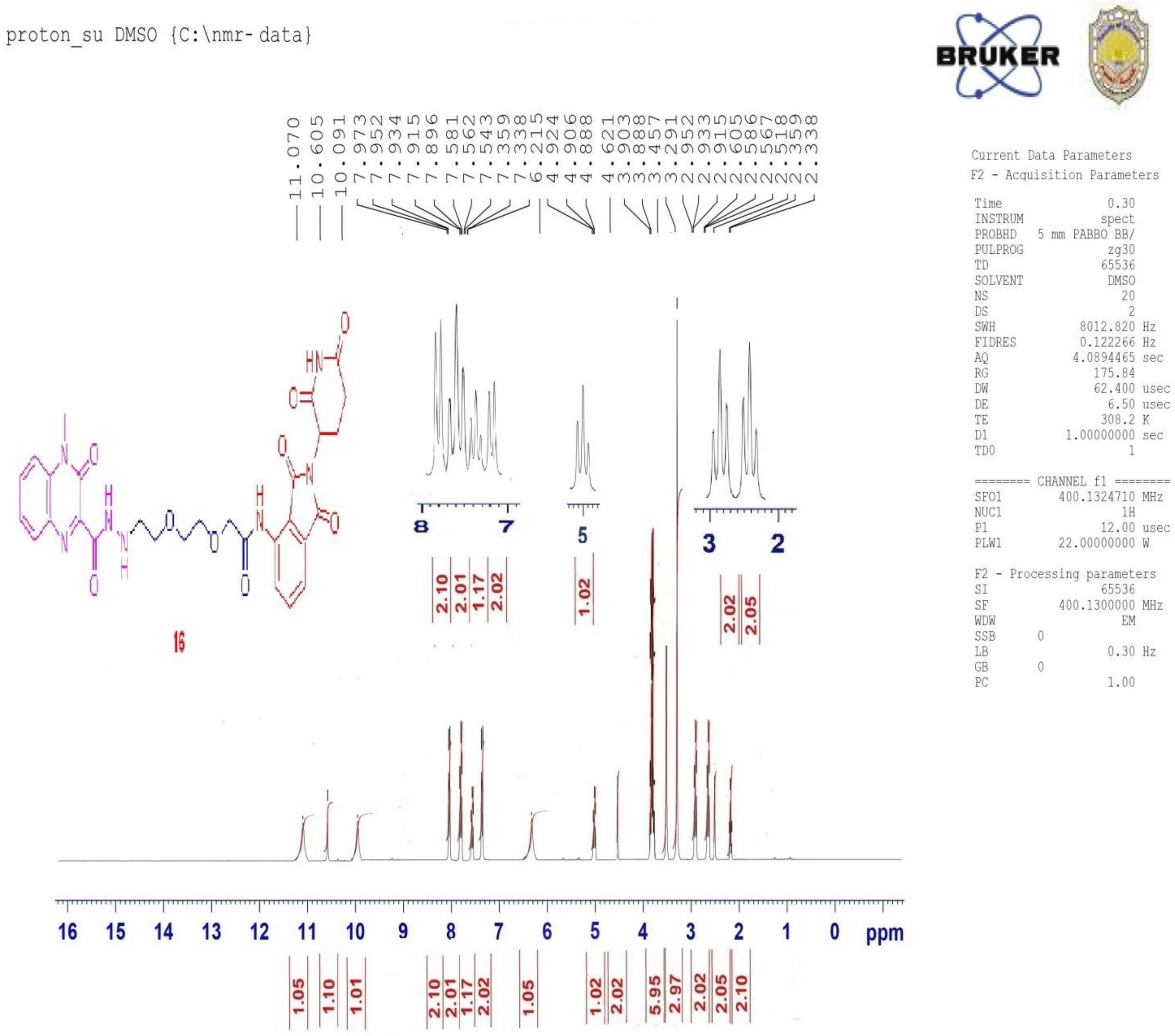

^{13}C NMR δ ppm

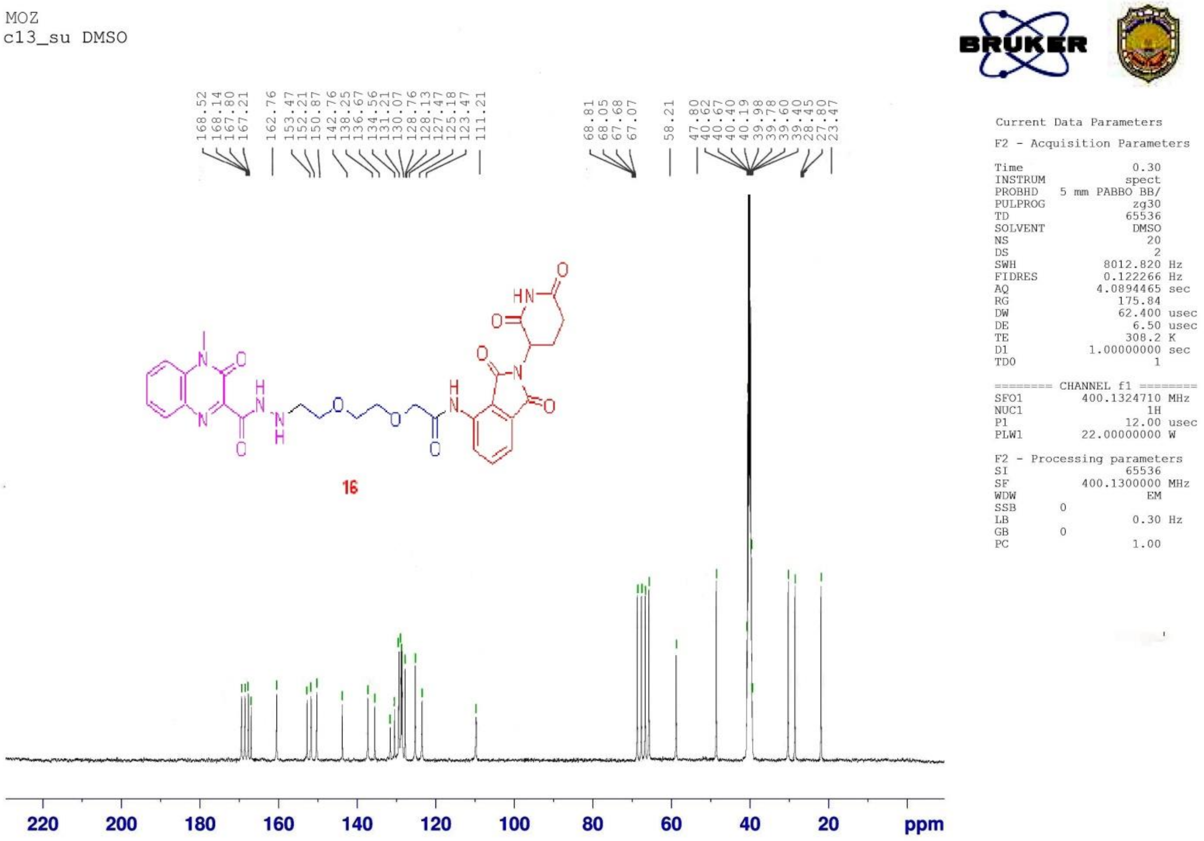

IR (KBr) ν cm^{-1}

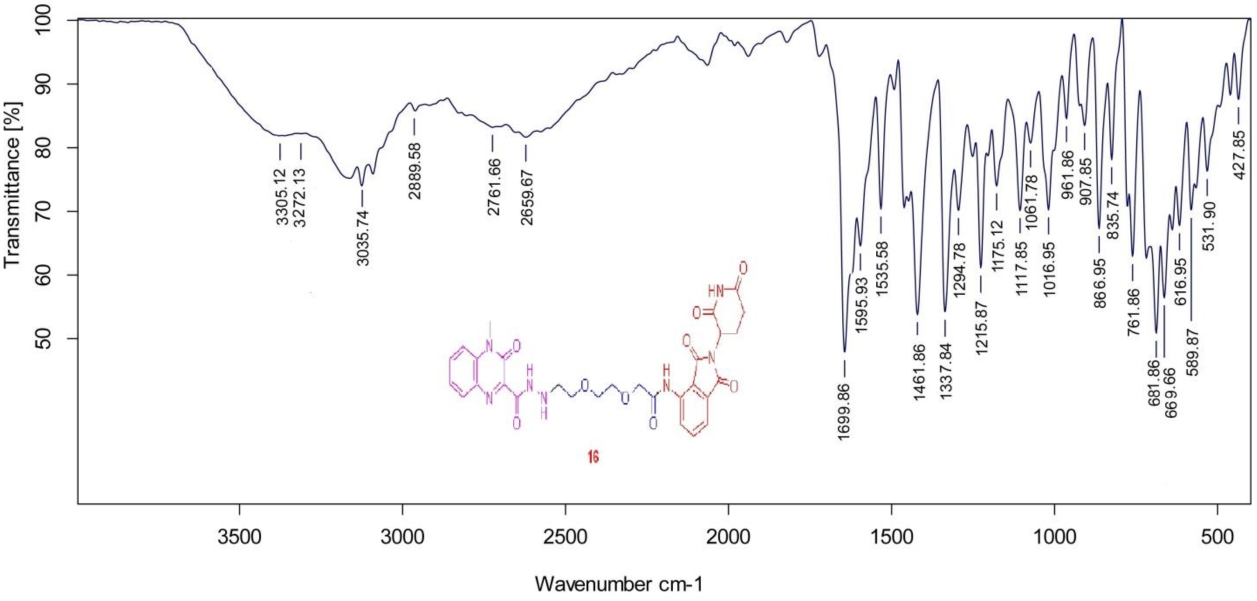
Transmittance [%]
100
90
80
70
60
50
3305.12
3272.13
3035.74
2889.58
2761.66
2659.67
1699.86
1595.93
1535.58
1461.86
1337.84
1294.78
1215.87
1175.12
1117.85
1061.78
1016.95
961.86
907.85
866.95
835.74
761.86
681.86
669.66
616.95
589.87
531.90
427.85
16
3500
3000
2500
2000
1500
1000
500
Wavenumber cm-1

3.1.2.3.*N*-(2-(2-(2-(2-(2-(2,6-dioxopiperidin-3-yl)-1,3-dioxoisoindolin-4-ylamino)-2-oxoethoxy)ethoxy)ethoxy)ethyl)-3-oxo-3,4-dihydroquinoxaline-2-carboxamide 17.

^{1}H NMR δ ppm

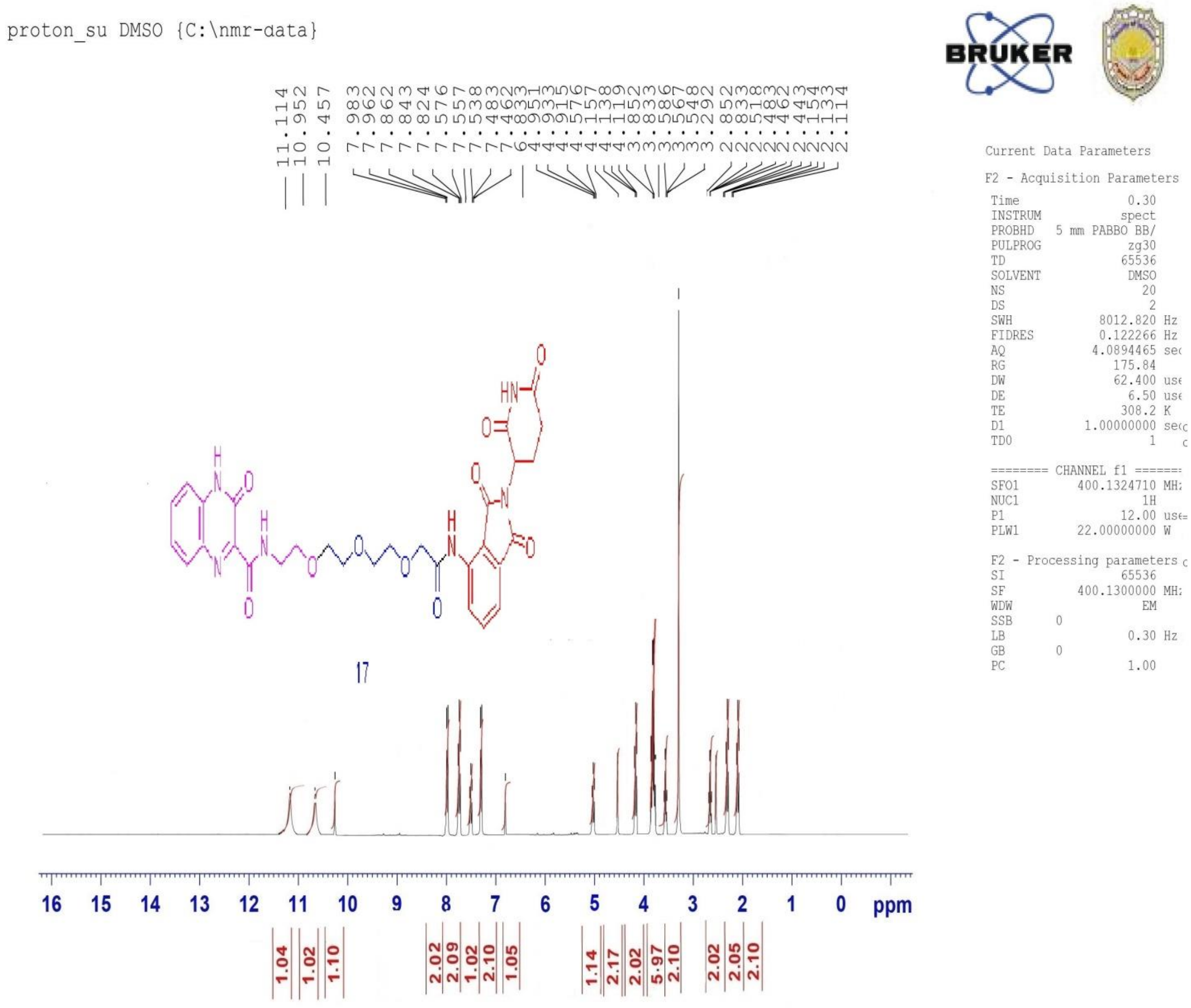

^{13}C NMR δ ppm

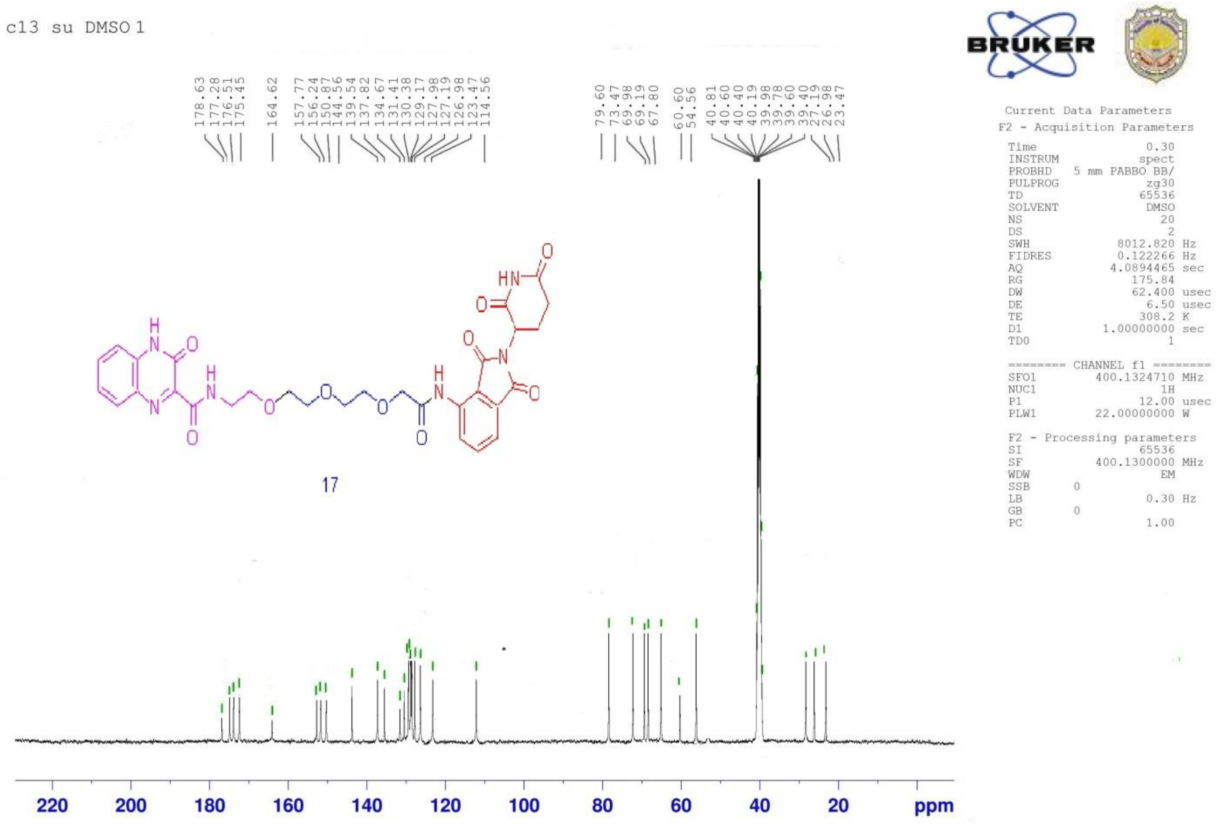

IR (KBr) ν cm^{-1}

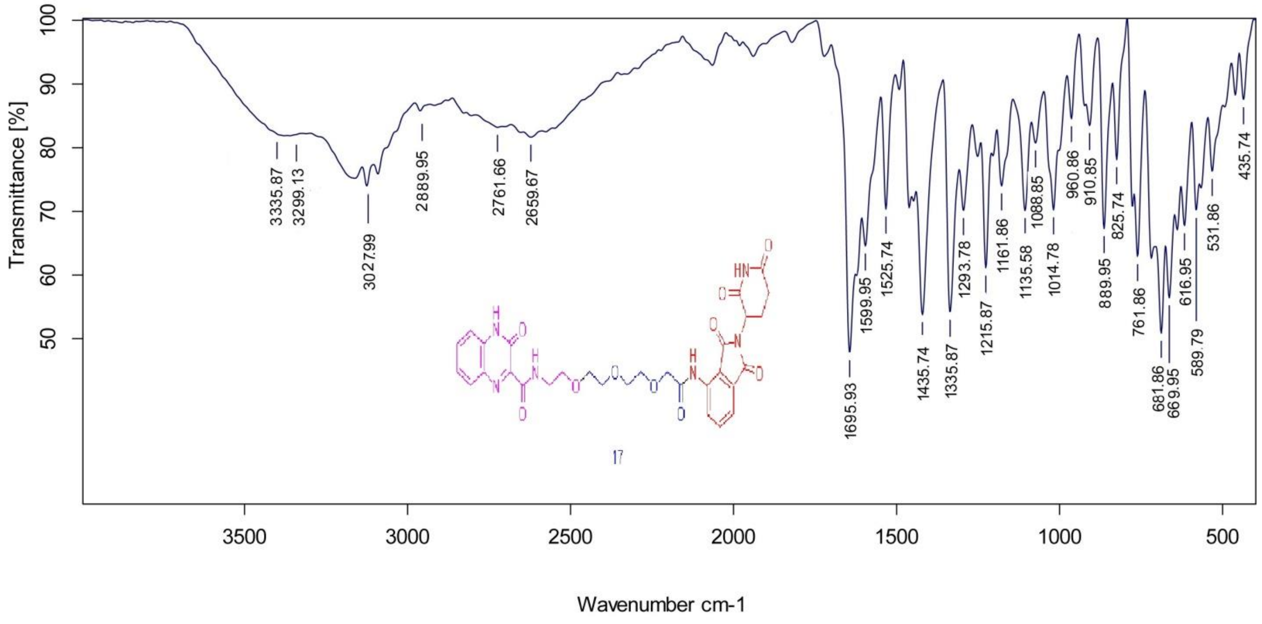
BRUKER
Transmittance [%]
Wavenumber cm-1
3335.87
3299.13
3027.99
2889.95
2761.66
2659.67
1695.93
1599.95
1525.74
1435.74
1335.87
1293.78
1215.87
1161.86
1135.58
1088.85
1014.78
960.86
910.85
889.95
825.74
761.86
681.86
669.95
616.95
589.79
531.86
435.74
17

3.1.2.4. *N*-(2-(2, 6-dioxopiperidin-3-yl)-1,3-dioxoisoindolin-4-yl)-2-(2-(2-(2-(4-((2-oxoindolin-3-ylidene)amino)benzoyl)hydrazineyl)ethoxy)ethoxy)acetamide 18.

^{1}H NMR δ ppm

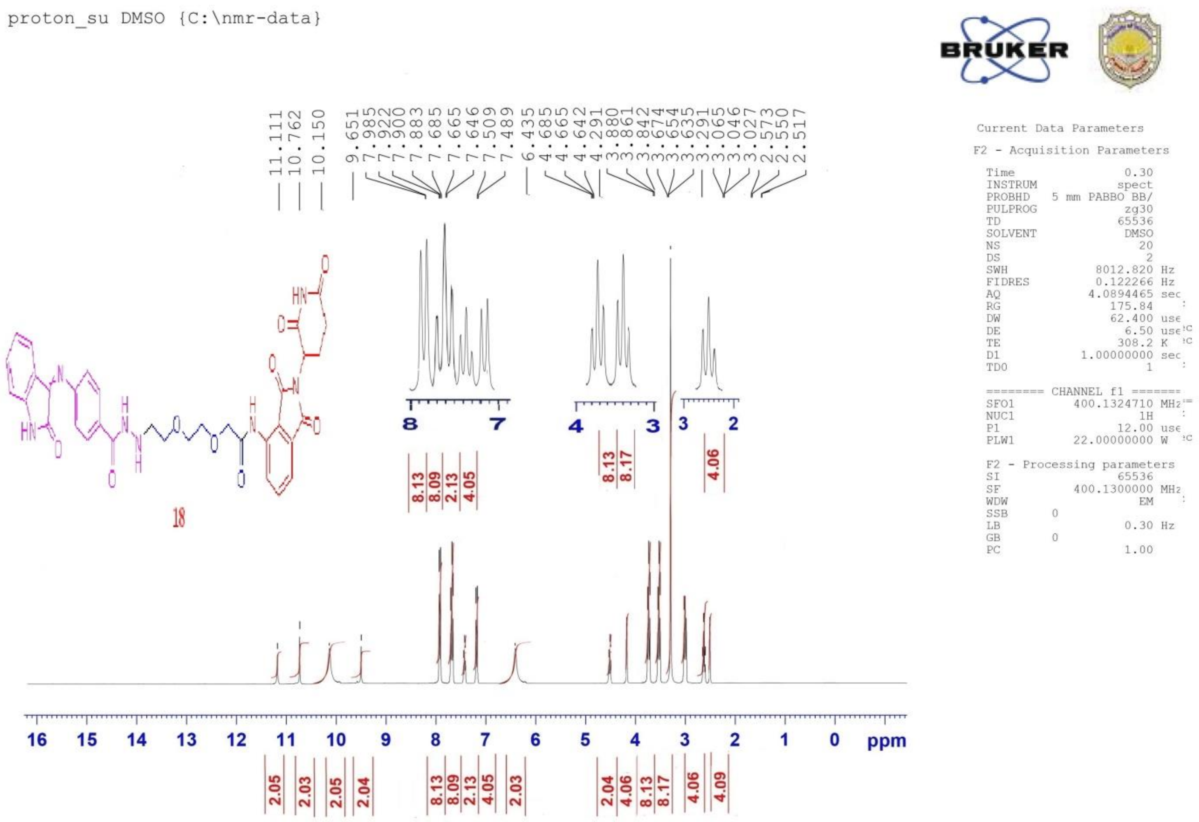

^{13}C NMR δ ppm

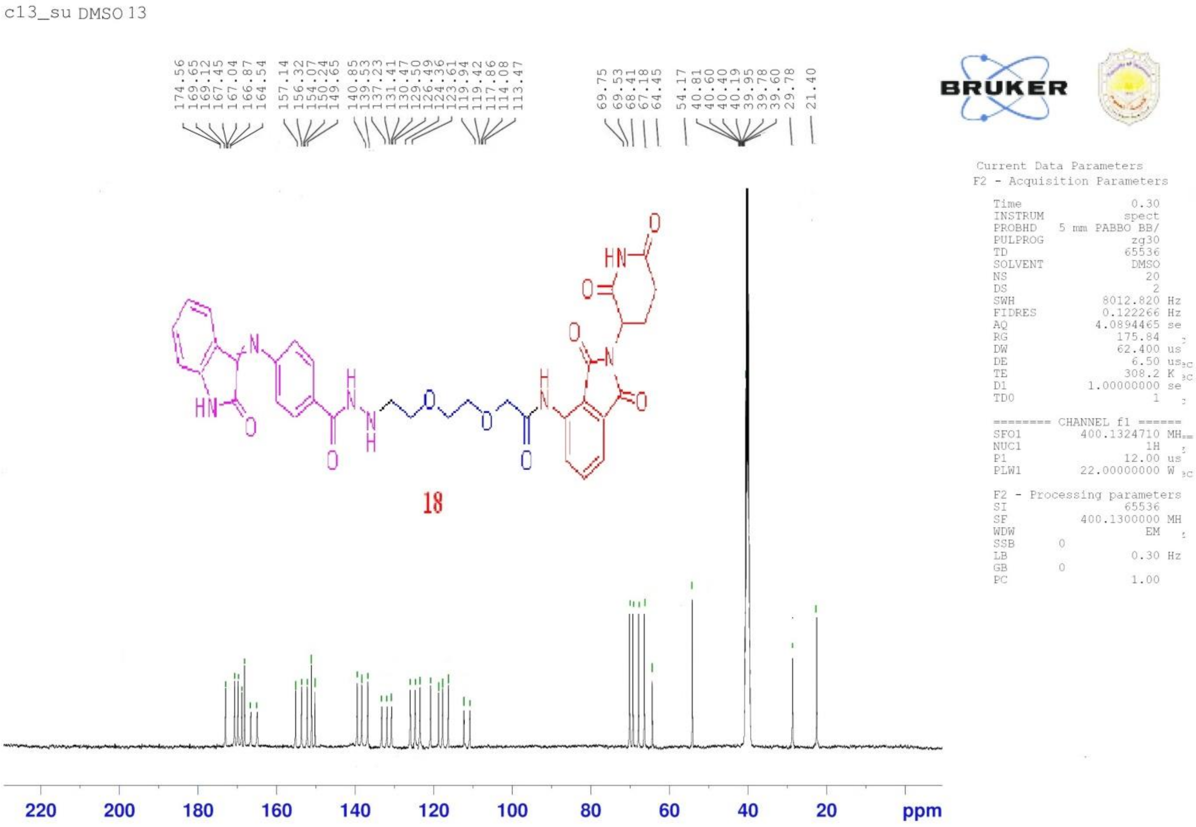

IR (KBr) ν cm^{-1}

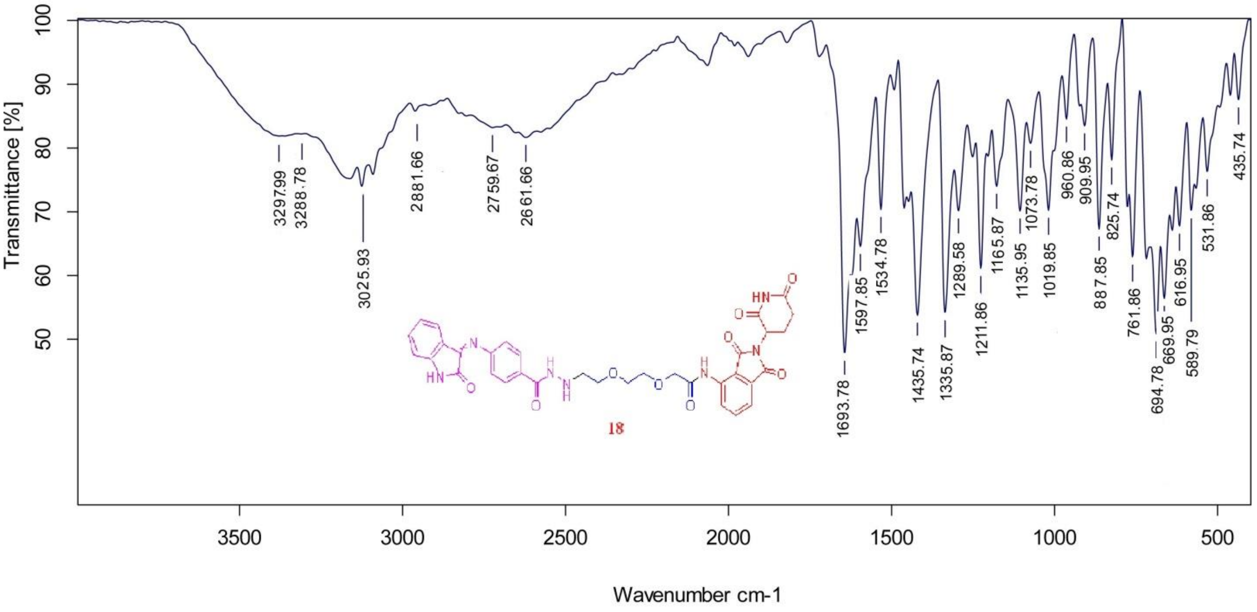

Transmittance [%]
Wavenumber cm-1
3297.99
3288.78
3025.93
2881.66
2759.67
2661.66
1693.78
1597.85
1534.78
1435.74
1335.87
1289.58
1211.86
1165.87
1135.95
1073.78
1019.85
960.86
909.95
887.85
825.74
761.86
694.78
669.95
616.95
589.79
531.86
435.74
18

3.1.2.5. *N*-(2-(2,6-dioxopiperidin-3-yl)-1,3-dioxoisoindolin-4-yl)-2-(2-(2-(2-(4-(1-methyl-2-oxoindolin-3-ylideneamino) benzoyl) hydrazinyl) ethoxy) ethoxy) acetamide 19.

^{1}H NMR δ ppm

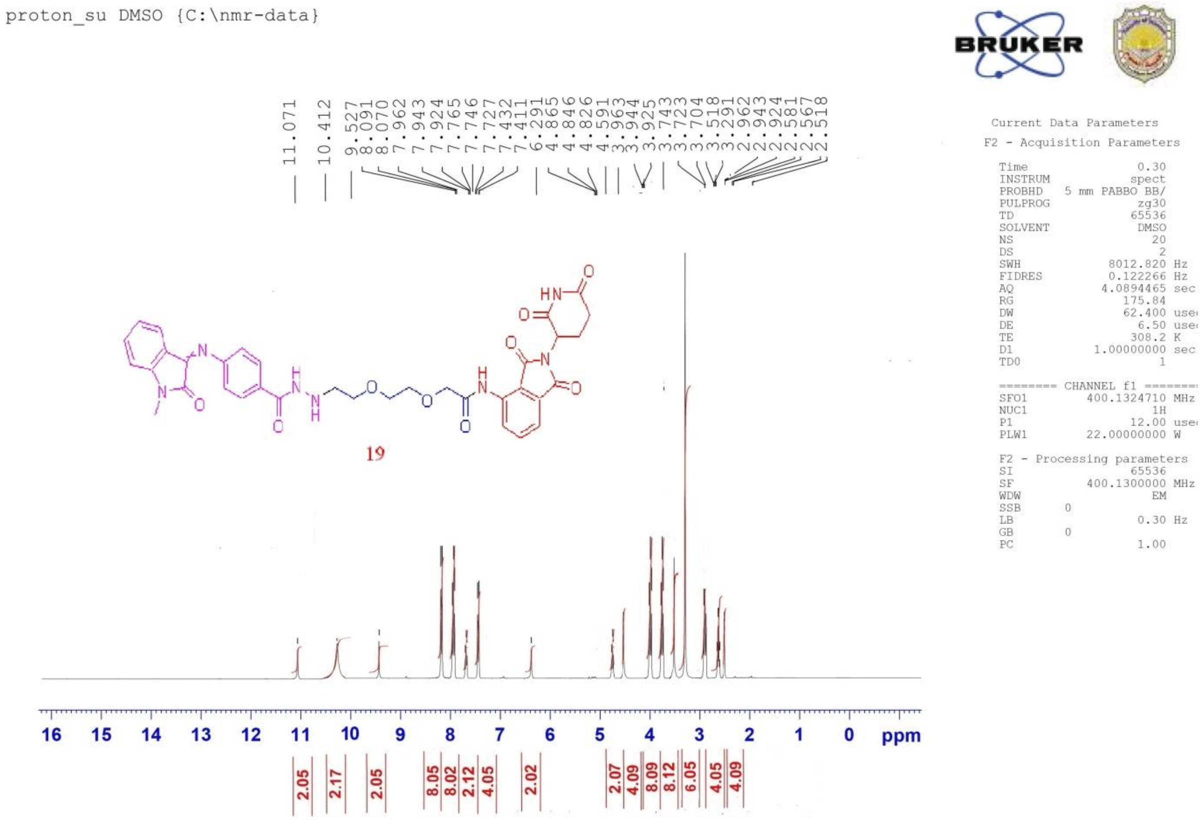

^{13}C NMR δ ppm

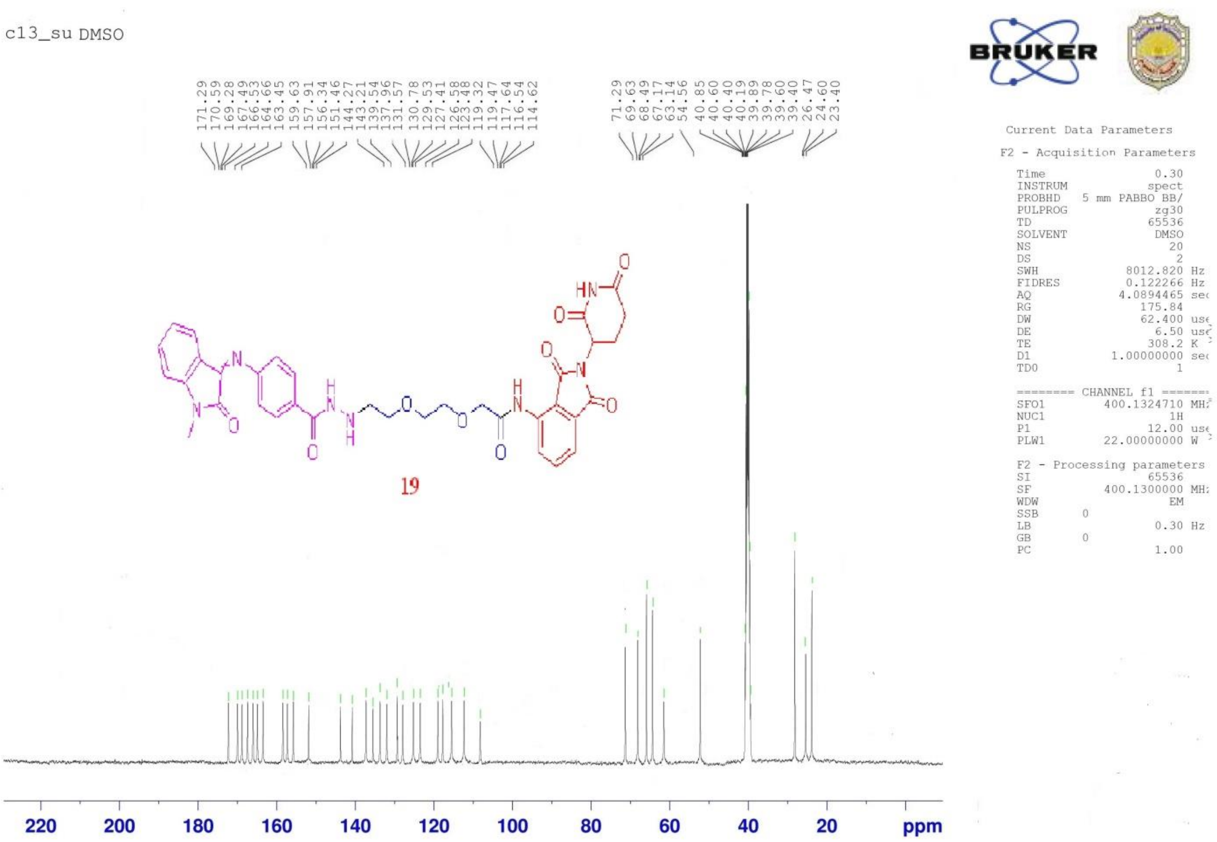

IR (KBr) ν cm^{-1}

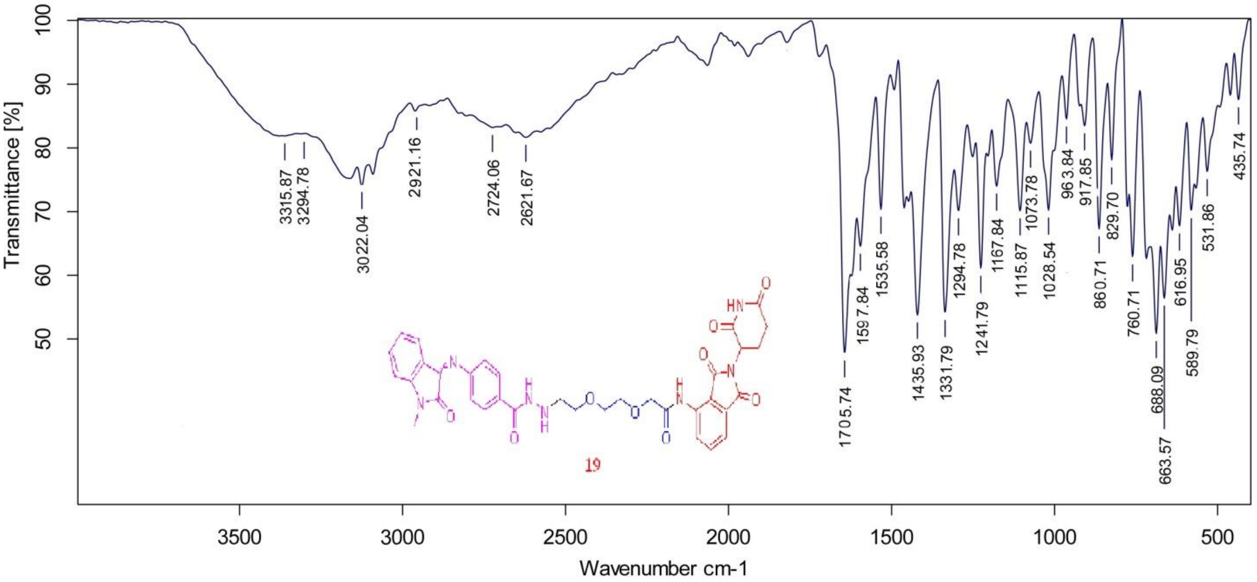

Sample Name: 100_ATR

3.1.2.6. 2-(2-(2-(2-(benzo[d]oxazol-2-ylthio) acetyl) hydrazinyl) ethoxy) ethoxy)-*N*-(2-(2, 6-dioxopiperidin-3-yl)-1, 3-dioxoisoindolin-4-yl) acetamide 20.

^{1}H NMR δ ppm

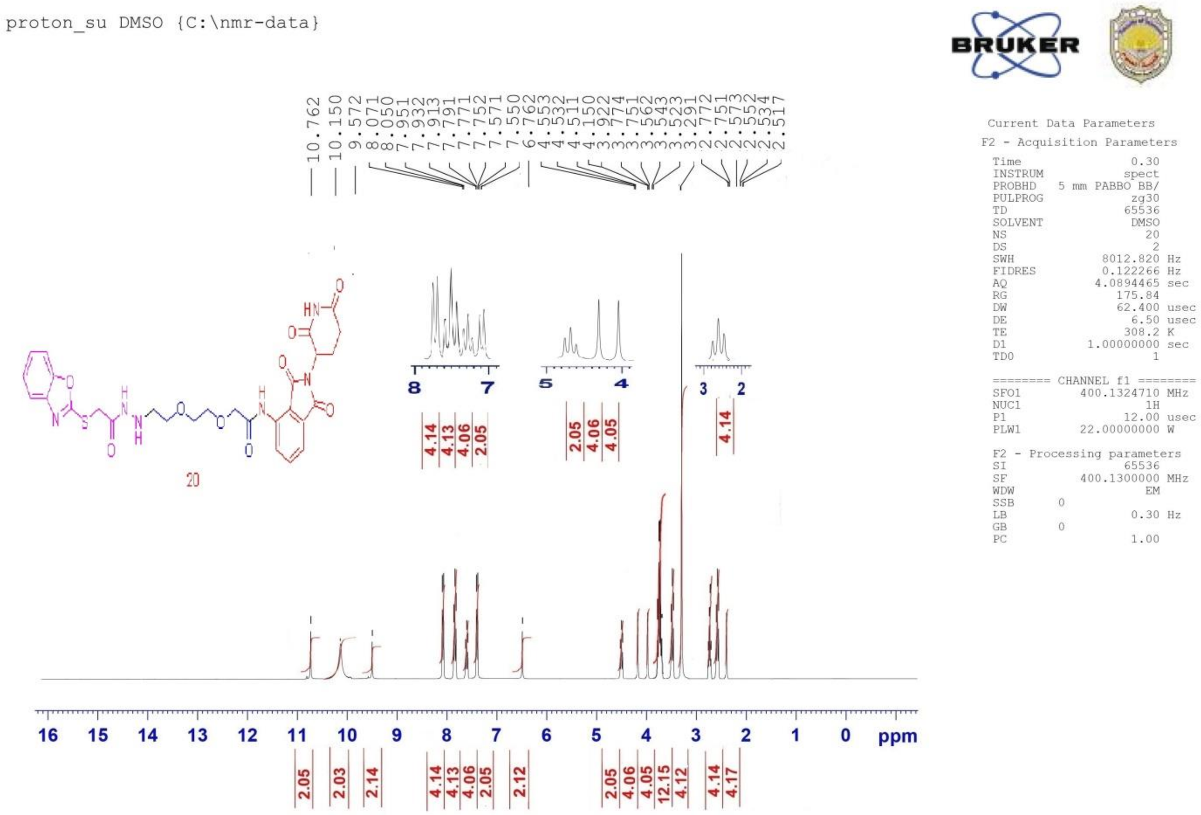

^{13}C NMR δ ppm

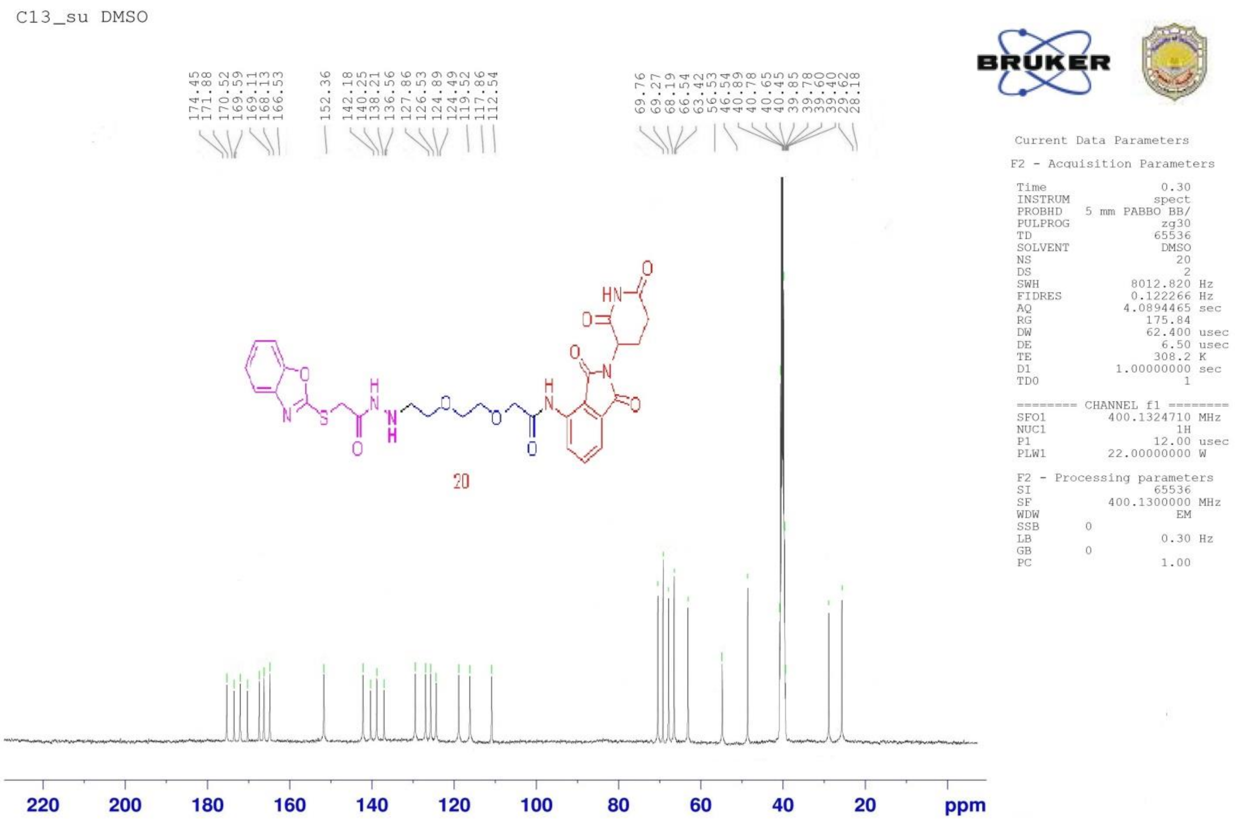

IR (KBr) ν cm^{-1}

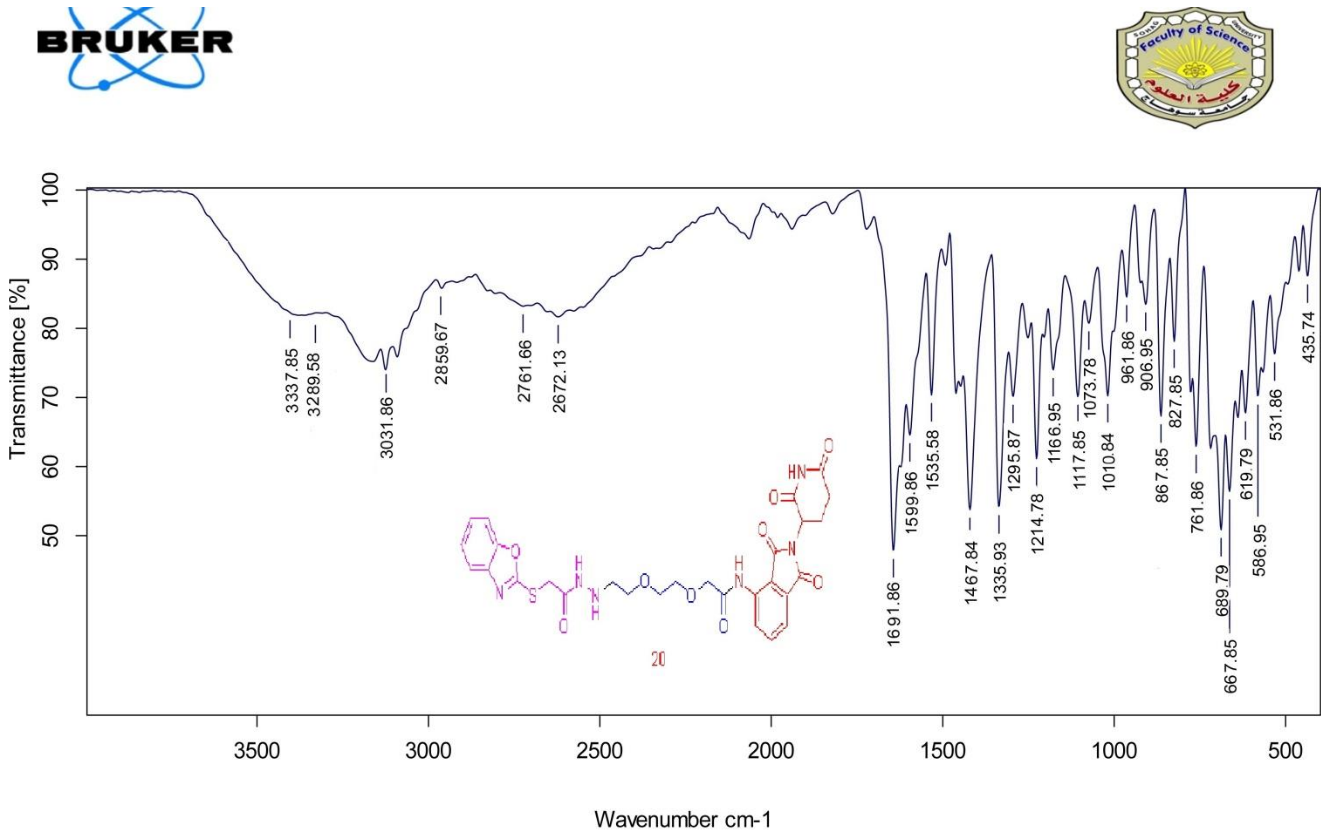
BRUKER
Transmittance [%]
Wavenumber cm-1
100
90
80
70
60
50
3500
3000
2500
2000
1500
1000
500
3337.85
3289.58
3031.86
2859.67
2761.66
2672.13
1691.86
1599.86
1535.58
1467.84
1335.93
1295.87
1214.78
1166.95
1117.85
1073.78
1010.84
961.86
906.95
867.85
827.85
761.86
689.79
667.85
619.79
586.95
531.86
435.74
20

3.1.2.7. 2-(2-(2-(2-(2-(benzo[d]thiazol-2-ylthio) acetyl) hydrazinyl) ethoxy) ethoxy)-*N*-(2-(2, 6-dioxopiperidin-3-yl)-1, 3-dioxoisoindolin-4-yl) acetamide 21.

^{1}H NMR δ ppm

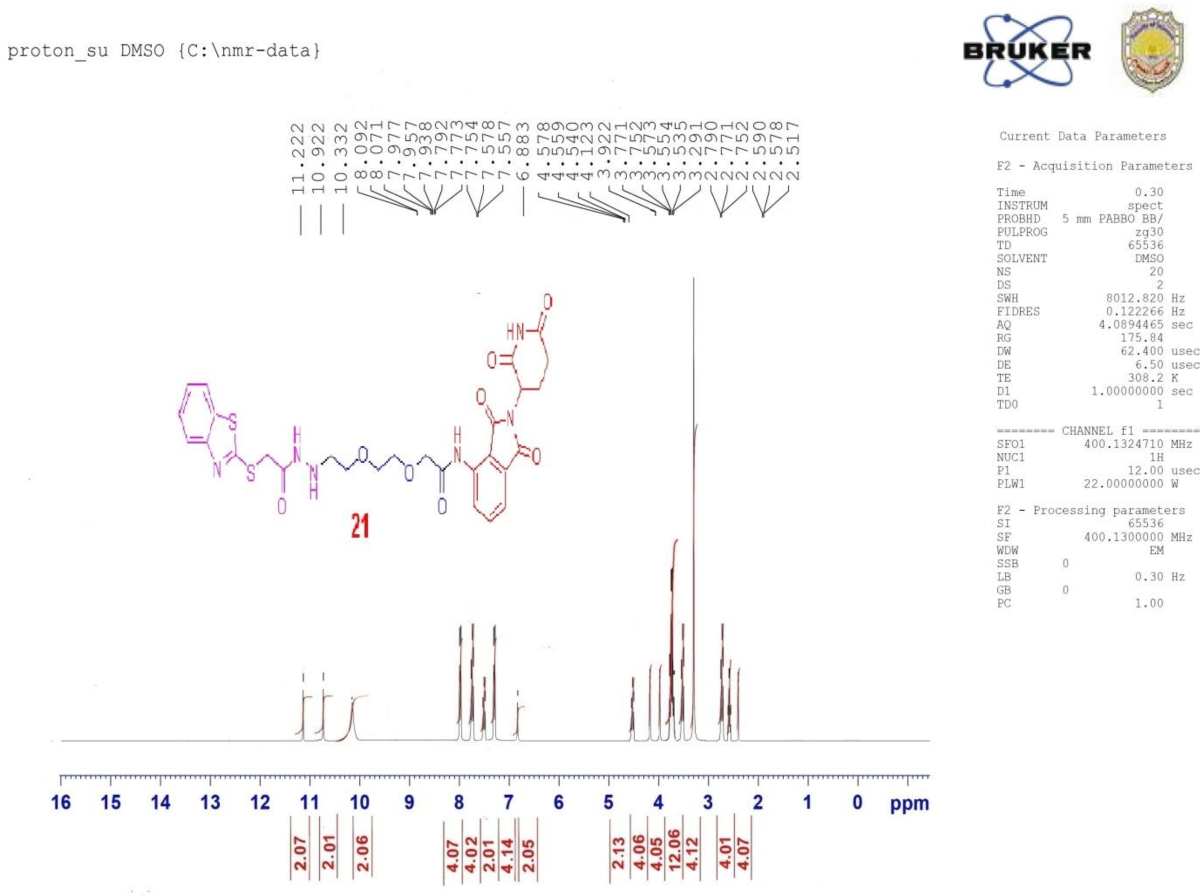

^{13}C NMR δ ppm

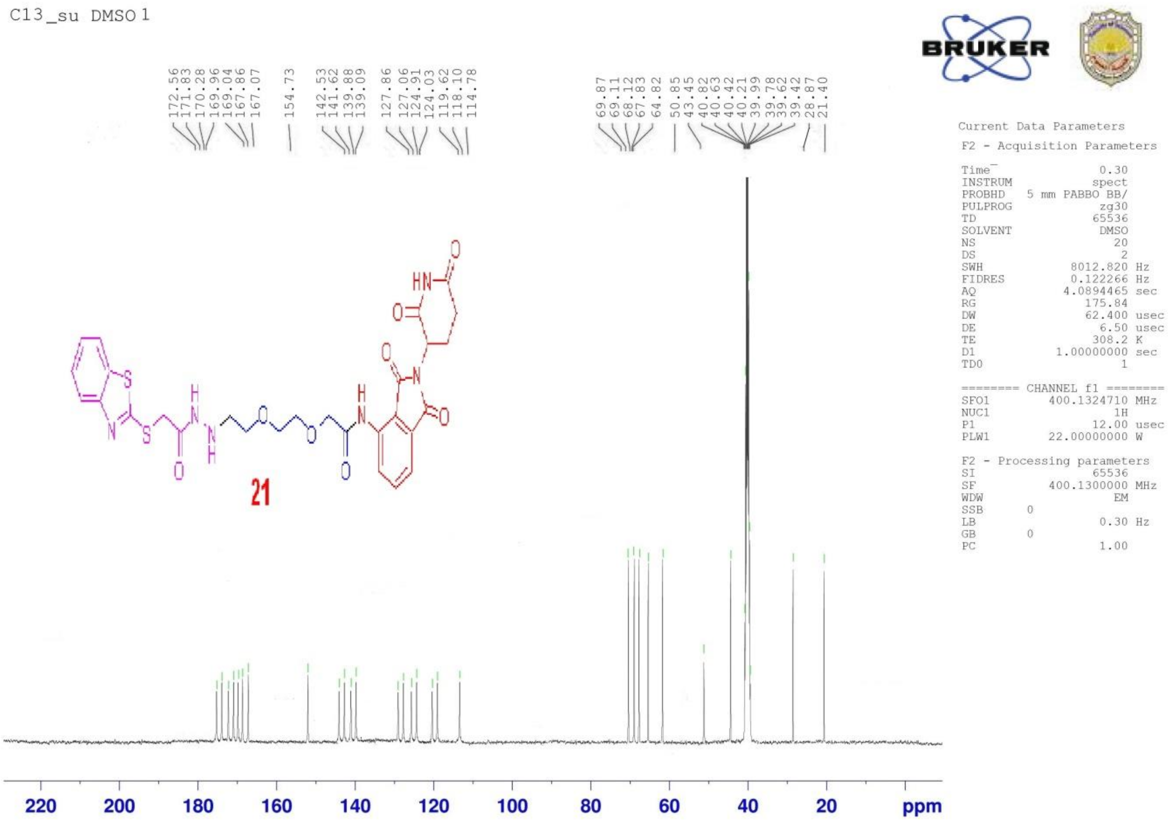

IR (KBr) ν cm^{-1}

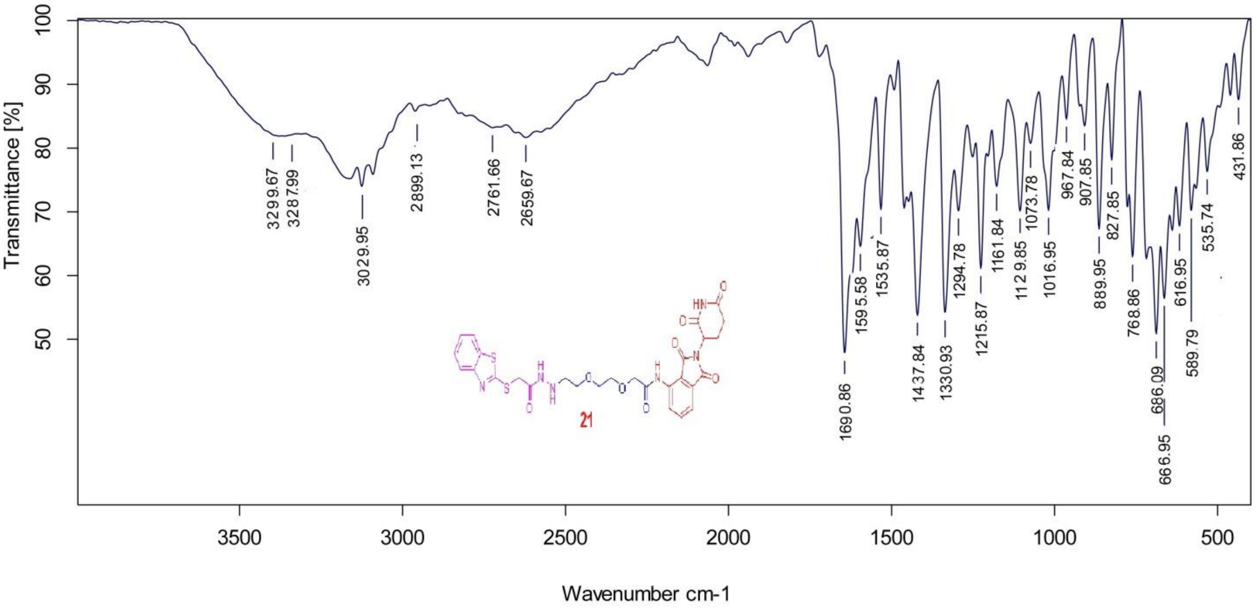

Transmittance [%]
100
90
80
70
60
50
3299.67
3287.99
3029.95
2899.13
2761.66
2659.67
1690.86
1595.58
1535.87
1437.84
1330.93
1294.78
1215.87
1161.84
1129.85
1073.78
1016.95
967.84
907.85
889.95
827.85
768.86
686.09
666.95
616.95
589.79
535.74
431.86
21
3500
3000
2500
2000
1500
1000
500
Wavenumber cm-1

. *2.3. In silico studies*

Molecular docking

A

B

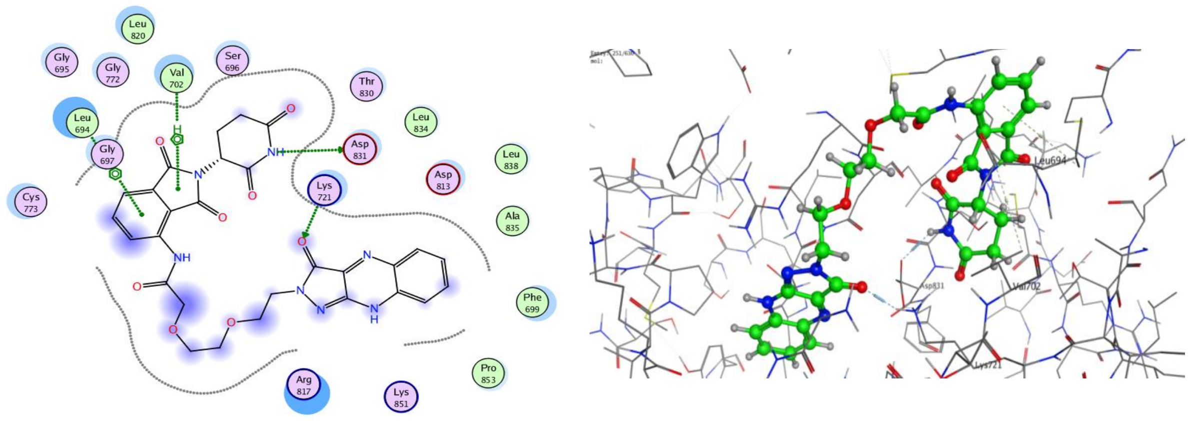

Figure 10. (A) and (B) 2D, 3D images of compound **15** docked in the EGFR^{WT} active site showing interactions with different amino acid residues found in the active site.

.

A

B

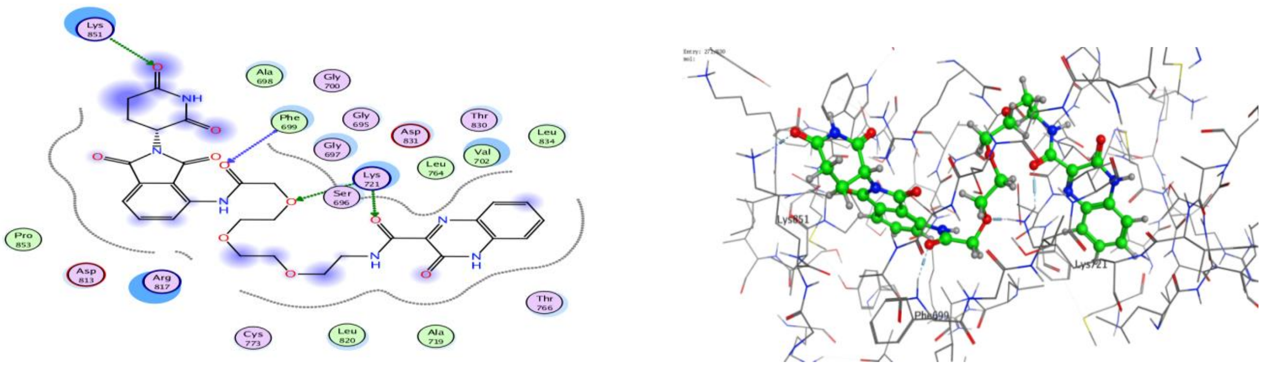

Figure 11. (A) and (B) 2D, 3D images of compound **17** docked in the EGFR^{WT} active site showing interactions with different amino acid residues found in the active site.

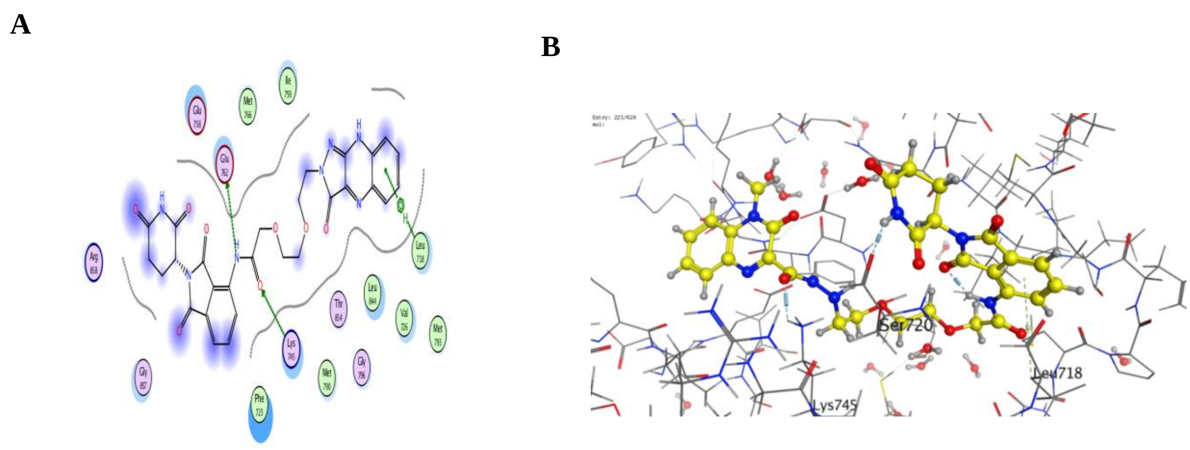

Figure 12. (A) and (B) 2D, 3D images of compound **15** docked into the active site of $EGFR^{T790M}$ showing interactions with different amino acid residues found in the active site.

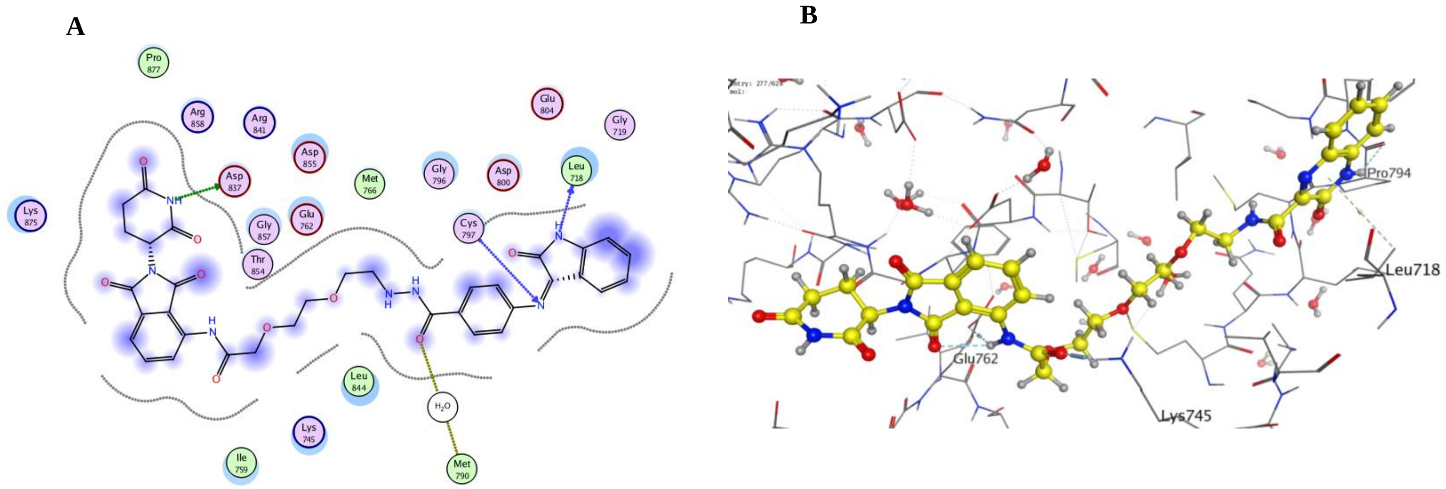

Figure 13. (A) and (B) 2D, 3D images of compound **17** docked in the $EGFR^{WT}$ active site showing interactions with different amino acid residues found in the active site.

3.3. In silico studies

Docking studies

The crystal structures of the target enzymes $EGFR^{WT}$ (PDB ID: 4HJO, resolution: 2.75 Å and $EGFR^{T790M}$ (PDB ID: 3W2O, resolution: 2.35 Å) were downloaded from the Protein Data Bank (http://www.pdb.org). Molecular Operating Environment (MOE) was used for the docking analysis. In these studies, the free energies and binding modes of the designed molecules against $EGFR^{WT}$ and $EGFR^{T790M}$ were determined. At first, the water molecules were removed from the crystal structures of $EGFR^{WT}$ and $EGFR^{T790M}$, retaining only one chain in each enzyme. Erlotinib and TAK-285 (the co-crystallized ligands) were utilized as references in the docking processes against both $EGFR^{WT}$ and $EGFR^{T790M}$, respectively. After that, in order to prepare the target molecules for binding with the designed compounds, the target proteins were subjected to a protonation step. Then, the hydrogen atoms were hidden to make the areas of interaction clearer. Next, the energy of all systems were minimized followed by identification of the binding pockets of the target proteins. The structures of the designed compounds and the co-crystallized ligands, erlotinib and TAK-285, were drawn using ChemBioDraw Ultra 14.0 and saved as an SDF format. Then, the saved files were opened using MOE and 3D structures were protonated. Next, the energy of the molecules was minimized. The validation process was performed for each target by running the docking process for only the co-crystallized ligand. Low RMSD values between docked and crystal conformations indicate valid performance. The docking procedures were carried out utilizing a default protocol. In each case, 7 docked structures were generated using genetic algorithm searches. The obtained figures from the MOE were further analyzed and visualized using Discovery Studio 4.0 software
